# Identifying novel mechanisms of biallelic *TP53* loss refines poor outcome for patients with multiple myeloma

**DOI:** 10.1038/s41408-023-00919-2

**Published:** 2023-09-11

**Authors:** Enze Liu, Parvathi Sudha, Nathan Becker, Oumaima Jaouadi, Attaya Suvannasankha, Kelvin Lee, Rafat Abonour, Mohammad Abu Zaid, Brian A. Walker

**Affiliations:** 1 Melvin and Bren Simon Comprehensive Cancer Center, Division of Hematology and Oncology, School of Medicine, Indiana University, Indianapolis, IN USA; 2 Center for Computational Biology and Bioinformatics, School of Medicine, Indiana University, Indianapolis, IN USA

**Keywords:** Cancer genomics, Cancer genomics

## Abstract

Biallelic *TP53* inactivation is the most important high-risk factor associated with poor survival in multiple myeloma. Classical biallelic *TP53* inactivation has been defined as simultaneous mutation and copy number loss in most studies; however, numerous studies have demonstrated that other factors could lead to the inactivation of *TP53*. Here, we hypothesized that novel biallelic *TP53* inactivated samples existed in the multiple myeloma population. A random forest regression model that exploited an expression signature of 16 differentially expressed genes between classical biallelic *TP53* and *TP53* wild-type samples was subsequently established and used to identify novel biallelic *TP53* samples from monoallelic *TP53* groups. The model reflected high accuracy and robust performance in newly diagnosed relapsed and refractory populations. Patient survival of classical and novel biallelic *TP53* samples was consistently much worse than those with mono-allelic or wild-type *TP53* status. We also demonstrated that some predicted biallelic *TP53* samples simultaneously had copy number loss and aberrant splicing, resulting in overexpression of high-risk transcript variants, leading to biallelic inactivation. We discovered that splice site mutation and overexpression of the splicing factor *MED18* were reasons for aberrant splicing. Taken together, our study unveiled the complex transcriptome of *TP53*, some of which might benefit future studies targeting abnormal *TP53*.

## Introduction

Multiple myeloma is a heterogeneous disease characterized by genomic markers that are used to delineate high-risk disease. There are many algorithms to determine how to define high-risk myeloma, and the genomic markers include t(4;14), t(14;16), gain 1q21 (*CKS1B*), del 1p32 (*CDKN2C*), and del 17p13 (*TP53*) [1–4]. Of these markers, *TP53* is probably the most important and is associated with rapid progression and poor overall survival.

Deletion of 17p has been seen as a poor prognostic marker since its discovery in MM [5]. Del 17p has been detected using fluorescence in situ hybridization, and although detection of deletion in as low as 10% of cells is associated with poor outcome [6], the larger the proportion of cells with loss of 17p, the stronger the effect on outcome [7, 8]. More recently, the use of molecular technologies has highlighted the importance of multi-hit or biallelic *TP53* abnormalities in MM. We have previously shown that biallelic abnormalities of *TP53*, comprising deletion and/or mutation of both alleles, are associated with outcome, whereas deletion alone is not [9]. The frequency of biallelic loss of *TP53* in MM increases with disease progression, being rare in smoldering myeloma (1.2%) and increasing through relapse (20%), indicating that it is a key mechanism in the pathogenesis of the disease [10, 11]. Biallelic loss of *TP53* has also been shown to be responsible for poor outcomes in myelodysplastic syndromes, myelofibrosis, and acute myeloid leukemia, pointing to a consistent abnormality in hematological malignancies [12, 13].

In addition to mutation and deletion of *TP53*, other mechanisms are at play that result in loss of cellular function of p53, including alternative splicing, promoter methylation, protein isoform usage, and changes in expression of gene regulators [14, 15]. These distinct mechanisms that result in the loss of functional p53 are currently impossible to determine solely at the DNA level but could be assessed by modeling the downstream transcriptomic signature of biallelic *TP53*.

Here, we utilized 634 newly diagnosed (NDMM) and 66 relapsed/refractory multiple myeloma (RRMM) samples from the MMRF CoMMpass dataset. By training a random forest regression model with transcriptomic features from known biallelic and wild-type samples, we predicted potential biallelic *TP53* samples from known monoallelic populations. Moreover, we demonstrated that predicted biallelic samples underwent expression of high-risk transcript variants and aberrant splicing but also investigated the reasons that led to them.

## Methods

### Defining ‘known’ biallelic TP53 samples

Mutation and copy number variation calls were obtained from the MMRF CoMMpass dataset web portal (version IA18). Identified somatic mutations had agreements from at least three out of four mutation callers: Mutect2 [16], Strelka2 [17], Octopus [18], and LANCET [19]. Copy number variations (CNVs) were identified by GATK [20], where copy number amplification (log_2_-fold change (Log2FC) ≥ 0.8), gain (0.2–0.8), deletion (−0.2 to −2.0), and deep deletion (<−2.0) were defined.

B-allele frequency was estimated by GATK [20] and normalized to scale (0 to 0.5), with 0.5 corresponding to the balanced copy number and 0 corresponding to the complete loss of heterozygosity (LOH) between the major and minor alleles. We subsequently used <0.25 as the threshold to determine LOH status.

‘Known’ biallelic inactivation of *TP53* (‘known’ biallelic) samples were defined as samples with either deep deletion, mutation plus deletion, or mutation plus LOH. Monoallelic *TP53* (‘known’ monoallelic) samples were defined as samples with a *TP53* mono-allelic mutation/CNV or LOH.

In total, 634 newly diagnosed multiple myeloma (NDMM) samples with mutation and CNV annotations were utilized, including 23 (3.6%), 62 (9.8%), and 549 (86.5%) samples with known biallelic, monoallelic, and wild-type status of *TP53*, respectively. Relapsed and refractory MM (RRMM) patients who developed plasma cell leukemia at any relapsed stage were excluded. This resulted in 66 RRMM samples, including 4, 15, and 47 samples with known biallelic, monoallelic, and wild-type status of *TP53*, respectively.

### Differentially expressed gene analysis

Gene expression of NDMM, RRMM, and 5 normal bone marrow plasma cell (BMPC) samples was quantified by transcript-per-million (TPM) [21] using Salmon [22] (Quasi-mode mapping, validate map mode) and Gencode V35 hg38 Gentrome (a combination of HG38 genome and Gencode HG38 transcriptome V35) as a reference. Expression profiles were rescaled with Log_2_(TPM + 1) transformation. Differentially expressed genes (DEGs) were identified between known biallelic and wild-type *TP53* samples using LIMMA [23]. Significant DEGs were defined as FC > 1.4 or FC < 0.71, FDR < 0.05, and Log_2_(TPM + 1) > 1 in either group.

### Determining proliferation (PR) Group from gene expression

The PR expression subgroup was previously described [24] (Supplementary Fig. 1E), and in this dataset, a PR group of 26 samples was identified.

### Transcript variant and protein domain inference

The Scallop pipeline (https://github.com/Kingsford-Group/scallop) was used to identify and quantify novel transcript variants. Transcriptomes were assembled by Scallop [25], while novel transcripts were identified by Gffread and quantified by Salmon [22]. Corresponding transcripts were extracted from the genome and translated to peptide sequences using the Expasy database [26]. Conserved domains were identified from predicted peptide sequences using the NCBI conserved domain database [27].

### Pathway analysis

Gene overrepresentation analysis (ORA) was conducted using WebGestalt [28]. Pathway enrichment analysis was conducted using GSEA [29]. The pathway knowledge bases ‘Kyoto Encyclopedia of Genes and Genomes’ (KEGG) [30] and Wikipathways [31] were used. Single-sample level pathway analysis was conducted using GSVA [32] with a GSEA-defined hallmark pathway set. Only significantly dysregulated (*p* < 0.05) pathways were reported and plotted.

### Model performance metrics

True positive rate, false positive rate, precision, and recall were calculated and used to generate receiver operating characteristic (ROC) curves and precision-recall curves (PRC). The area under the ROC curve (AUROC) and area under the PRC (AUPRC) were subsequently generated to measure the overall performance of the models.

### Hyperparameter tuning

To obtain the optimal hyperparameters that resulted in the model with the best performance, an exhaustive ‘grid search’ in the ‘scikit-learn’ [33] package was conducted for the number of trees (*n* ∈ (1, Number of Genes)), the minimum number of samples required to be a leaf node (*n* ∈ (0, 1)) and minimum weight fraction of the sum total of weights required to be at a leaf node (*n* $\in$ (0, 1)) with an offset of 0.05. The model with the highest AUPRC and AUROC > 0.8 was selected.

### Survival analysis

The MMRF CoMMpass IA18 clinical annotation for 634 NDMM samples and 66 RRMM samples was utilized. The log-rank test and Cox regression tests were used to examine the survival difference between groups. Kaplan‒Meier curves were drawn to describe progression-free survival (PFS), overall survival (OS), and after-relapse survival (ARS). In this study, ARS was defined as the survival duration of patients after *TP53* abnormality was detected/predicted for the first time.

### Alternative splicing analysis

rMATS [34] was used to identify AS events in *TP53* regions between each MM patient and 5 normal bone marrow plasma cell (BMPC) samples. Percent-of-Spliced-In (PSI) was used to measure the splicing level per sample, while deltaPSI (dPSI) was used to measure average splicing differences between the two groups. The retention intron (RI) events were observed from Sashimi plots, and PSI and dPSI were calculated as follows:

$${\rm{PSI}}=\frac{{\rm{reads}}\,{\rm{on}}\,{\rm{the}}\,{\rm{intron}}}{{\rm{Junction}}\,{\rm{reads}}\,+\,{\rm{reads}}\,{\rm{on}}\,{\rm{the}}\,{\rm{intron}}}$$

$${\rm{dPSI}}={\rm{PSI}}_{\rm{normal}}^{-}-{\rm{PSI}}_{\rm{tumor}}^{-}$$

## Results

### Model training and validation

Although biallelic *TP53* is currently defined by DNA methodologies, which identify mutation and copy number loss, other mechanisms of inactivation can occur, including loss of expression, alternative splicing, and generation of rare protein isoforms. These additional mechanisms could be difficult to identify but may be inferred from modeling downstream expression signatures.

**Fig. 1:**
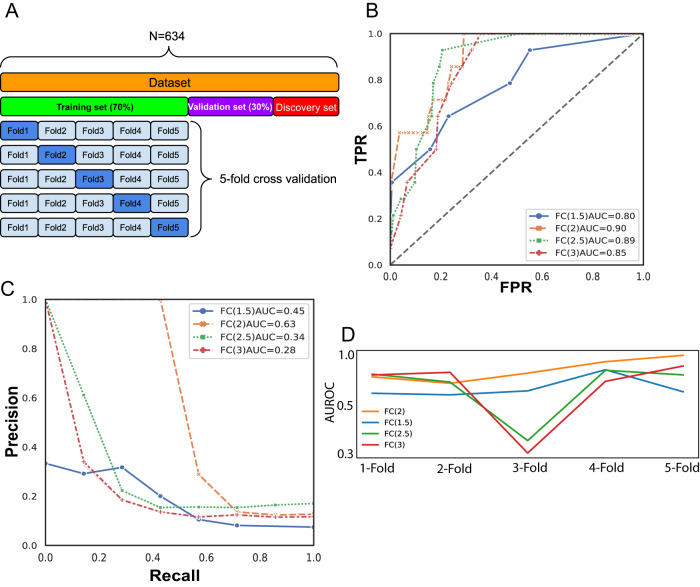
A random forest model for predicting biallelic *TP53* samples from gene expression profile. **A** Data composition for model training and validation. **B** Receiver operating characteristic (ROC) curves. **C** Precision–recall curves. **D** Fivefold cross-validation results measure the performance of the models with various sets of differentially expressed genes.

Using 634 NDMM samples with mutation and copy number annotations, we determined biallelic, monoallelic, and wild-type *TP53* status. Differentially expressed gene (DEG) analysis was conducted between existing known biallelic *TP53* (*n* = 23) and WT samples (*n* = 549) to identify a biallelic *TP53* expression signature, and samples were split into training and validation sets in a 7:3 ratio (Fig. 1A). DEGs that were either significantly (FDR < 0.05) up- or downregulated in biallelic *TP53* were defined based on the fold-change (FC), namely, ‘FC1.5’, ‘FC2’, ‘FC2.5’ and ‘FC3’ (Supplementary Fig. 1A). Random forest regression models were proposed to predict biallelic *TP53* samples from *TP53* wild-type (WT) samples using their expression profiles, and fivefold cross-validation was subsequently performed to measure the robustness of the established models (Fig. 1A).

The random forest regression models were established using each set of DEGs as features and compared by their area under precision-recall curves (AUPRCs) and area under receiver operative characteristics curves (AUROCs). A hyperparameter tuning method was conducted to identify the parameter set corresponding to the optimal performance. Given that the dataset is highly imbalanced (4.1% biallelic *TP53* samples in the population), precision-recall curves are more informative and accurate when measuring model performance [35, 36]. Hence, models with different parameters were prioritized by AUPRC first and subsequently by a high AUROC threshold (>0.8). Curves indicated models with the best performance after hyperparameter tuning (Fig. 1B, C). The optimal model was derived from the FC > 2 DEG set and reflected not only the highest AUPRC among all but also consistently high performance in 5-fold cross-validation (AUROC ∈ (0.82,1)). This model was subsequently selected for further analysis (Fig. 1D).

**Fig. 2:**
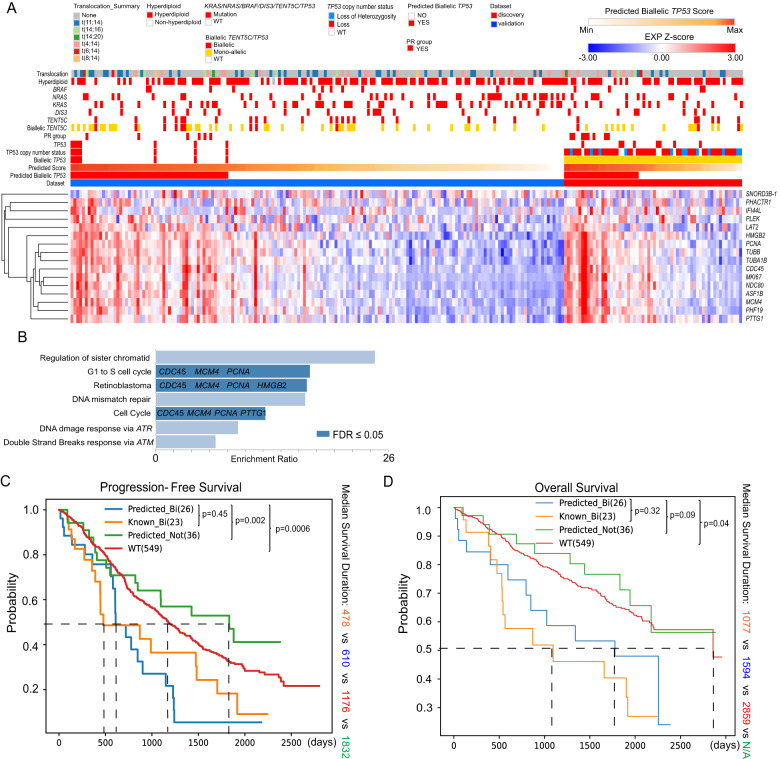
Characteristics of predicted biallelic samples compared to known biallelic samples. **A** Gene expression heatmap of 16 signature genes across NDMM samples in the validation and discovery sets. **B** Dysregulated pathways enriched by the 16 signature genes; Kaplan‒Meier survival curves for ‘known’ biallelic, ‘predicted’ biallelic, ‘predicted’ mono-allelic and ‘known’ wild-type *TP53* groups for progression-free survival (**C**) and overall survival (**D**). TPR true positive rate, FPR false positive rate, AUROC area under ROC.

The final model consisted of 25 trees, comprising 16 genes (Fig. 2A) out of 100 DEGs in the ‘FC2’ set (Supplementary Fig. 1B). Among the 16 genes, 14 were connected in a protein‒protein interaction (PPI) network (Supplementary Fig. 1C). Five genes were directly involved in the cell cycle, and related pathways (Fig. 2B). Of the other two genes, *NDC80* (previously known as Hec1) is a key regulator of G2/M phase [37] and is often overexpressed in human cancers, including MM [38]. High expression of *PHF19* has been reported [39] to be associated with high-risk disease in myeloma. Most of the significantly dysregulated pathways from 16 genes were related to the cell cycle (Fig. 2B), which is in line with the previously reported roles of *TP53* [40]. Individual-level pathway analysis was also conducted for all samples using GSVA and hallmark cancer pathway sets defined by the GSEA database. The top dysregulated pathways between known biallelic and WT samples included *MYC* targets, cell cycle, DNA repair, and oxidative phosphorylation, and these pathways were consistently upregulated between known and predicted biallelic samples (Supplementary Fig. 1D).

### Characteristics of predicted novel biallelic samples in NDMM

Samples in the validation dataset were given a predicted score (Fig. 2A). To reach the maximum sensitivity, a cutoff of the predicted score was set until the last known biallelic sample was included, resulting in 100% sensitivity and 71.4% specificity. We noted the enrichment of several genomic markers in the predicted biallelic *TP53* samples, including biallelic *TENT5C* (*N* = 6; *p* = 0.03, chi-square test), suggesting a similar regulation of gene expression between biallelic *TENT5C* and *TP53* alterations. There was also enrichment for the PR subgroup expression signature [24] (*N* = 6; *p* = 0.002, chi-square test) in the predicted biallelic *TP53* samples, indicating a similarity between the PR signature and the biallelic *TP53* signature, suggesting similar mechanisms at action.

### Novel biallelic *TP53* samples have a similar expression profile and are associated with a poor outcome

We hypothesize that there will be more novel biallelic *TP53* samples in those that are already monoallelic defined by mutation or deletion. The established model was therefore used to predict novel biallelic samples from known monoallelic samples, referred to as the discovery set (Table 1). The same cutoff score was applied to the discovery set of known monoallelic *TP53* samples (*N* = 62), leading to the identification of 26 (42%) predicted biallelic samples. At the gene level, 26 samples were expressing *TP53* (Log_2_(TPM + 1) > 1). Differential expression analysis between the 26 predicted and 23 known biallelic samples identified no significantly dysregulated genes, indicating parity between the two sets. In contrast, 939 significant DEGs were identified between the 26 predicted biallelic and 36 confirmed monoallelic samples from the discovery set. Subsequent GSEA analysis identified significantly upregulated KEGG pathways covering the cell cycle/DNA replication, DNA damage repair, and p53 signaling (Supplementary Fig. 2A). These upregulated categories were consistently observed from the same GSEA between the 23 known biallelic and 36 predicted monoallelic samples (Supplementary Fig. 2B).

**Table 1:** Number of samples and their *TP53* abnormalities.

State	Set	Group	TP53 abnormalities (*N* =)			
			Mutation	CNV	LOH	Total
NDMM (*N* = 634)	Training	Known biallelic	16	15	1	16
		Known WT				377
	Validation	Known biallelic	7	7	0	7
		predicted as biallelic				7
		predicted not				0
		Known WT				165
		predicted as biallelic				48
		predicted not				117
	Discovery	Known mono-allelic	8	42	12	62
		predicted as biallelic	4	18	4	26
		predicted not	4	24	8	36
RRMM (*N* = 66)	Validation	Known biallelic	4	4	0	4
		predicted as biallelic				4
		predicted not				0
		Known WT				47
		predicted as biallelic				25
		predicted not				22
	Discovery	Known mono-allelic	4	11	0	15
		predicted as biallelic	2	5	0	7
		predicted not	2	6	0	8

Additionally, we compared the number of SV events in known biallelic, predicted biallelic, predicted monoallelic, and WT groups (Supplementary Fig. 2C). No significant difference was found between the known and predicted groups (median 48 vs. 31, *p* = 0.23, Mann‒Whitney U test), while both groups contained significantly more events than the WT group (median 48 vs. 31 vs. 22, *p* = 0.0002 and *p* = 0.04). This fact still held when both groups were compared against the predicted monoallelic group (median 48 vs. 31 vs. 28, *p* = 0.01 and *p* = 0.07).

Patient survival was compared between the 23 known biallelic, 26 predicted biallelic, 36 confirmed monoallelic, and 549 wild-type samples. As expected, the previously defined monoallelic group (*N* = 62) was not associated with a different outcome compared to WT samples, while known biallelic samples (*N* = 23) were associated with significantly worse survival than both groups in PFS (median survival (days): 478 vs. 900 vs. 1176, *p* = 0.17 and *p* = 0.02, log-rank test) and OS (median survival (days): 1094 vs. 2256 vs. 2859, *p* = 0.02 and *p* = 0.003, Supplementary Fig. 2D, E). However, using the new categorization, the predicted biallelic samples were associated with a significantly worse outcome than the predicted monoallelic and WT samples with PFS (median survival (days): 623 vs. 1832 vs. 1176, *p* = 0.002 and *p* = 0.0006) and OS (median survival (days): 1794 vs. not reached vs. 2859, *p* = 0.09 and *p* = 0.04) (Fig. 2C, D). Moreover, the survival difference between the confirmed monoallelic group and WT was not significant for PFS (*p* = 0.2) and OS (*p* = 0.6) (Fig. 2C, D). Conversely, the predicted biallelic group showed no significant survival difference compared to the known biallelic group for PFS and OS, indicating a similar patient outcome between known and predicted biallelic samples. When known biallelic and predicted biallelic samples were combined in one group (*N* = 49), their survival remained significantly worse than the confirmed monoallelic and WT samples for PFS (median survival (days): 610 vs. 1176 vs. 1832, *p* = 0.002 and *p* = 0.0001) and OS (median survival (days): 1340 vs. not-reached vs. 2859, *p* = 0.01 and *p* = 0.0002, Supplementary Fig. 2F, G).

### The model predicts novel biallelic *TP53* samples from relapsed or refractory multiple myeloma (RRMM)

**Fig. 3:**
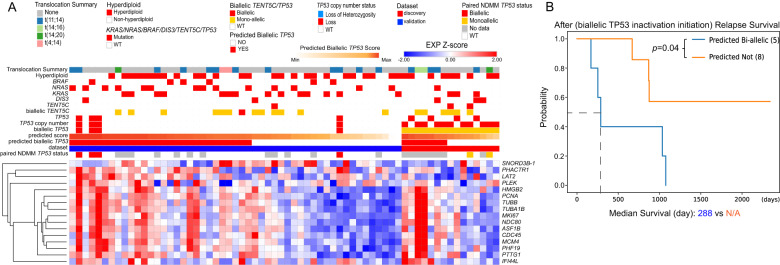
The model predicts biallelic *TP53* samples in relapsed and refractory multiple myeloma samples. **A** Gene expression heatmap of the 16 signature genes in RRMM samples in the validation and discovery datasets. **B** Difference in after-relapse survival between predicted biallelic and non-biallelic samples in the discovery set.

To explore the prediction power further, the model was applied to 66 RRMM samples (Table 1). Four known biallelic samples existed in the validation set (Fig. 3A), and their corresponding NDMM samples from the same patients were also biallelic. The model resulted in a 76% AUROC and 42% AUPRC from the validation set of four known biallelic and 47 wild-type samples, indicating consistently good performance. With the same threshold applied as in the NDMM population, 75% sensitivity (3/4) and 47% specificity (22/47) were achieved. The model predicted seven biallelic samples that corresponded to 5 patients from 15 monoallelic samples in the discovery set (Fig. 3A). Among them, four monoallelic samples from two patients were consistently predicted as biallelic samples. All seven samples were expressing *TP53* (Log2(TPM + 1) > 1).

After-relapse survival analysis conducted between the five predicted biallelic and the eight remaining monoallelic patients in the discovery set showed that the patients with predicted biallelic *TP53* had a significantly inferior after-relapse survival (*p* = 0.04, log-rank test) compared to the other eight monoallelic *TP53* patients (Fig. 3B). Meanwhile, the duration before such relapse was not significantly different (Supplementary Fig. 3). Taken together, this observation is in line with the inferior survival of patients once biallelic *TP53* inactivation was detected.

### Aberrant splicing and transcript variant expression were found in predicted biallelic *TP53* samples

*TP53* has numerous transcripts, some of which encode isoforms that deviate from the original tumor suppressor role and offer unique functions under different contexts [41]. Previous studies identified three major *TP53* protein isoforms (*α*, *β*, and *γ*), each of which had four different lengths (full length (TA), ∆40, ∆133, and ∆160) [15, 41]. In MM, patients with high expression of ${\rm{TAp53}}\beta \,{\rm{and}}\,{\rm{TAp}}53\gamma$ isoforms were associated with significantly worse survival than patients without [15]. Conversely, high expression of ∆133 and ∆160 was associated with a good prognosis [15]. We speculated that there was an expression of adverse *TP53* transcripts that, along with mutations or copy number alterations, led to its complete loss of function and would identify additional biallelic *TP53* samples that would not be detected by DNA techniques alone.

**Fig. 4:**
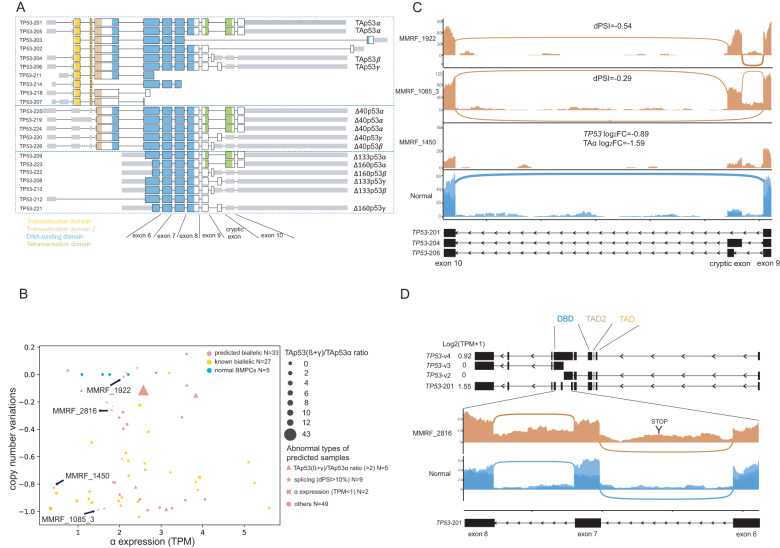
High-risk *TP53* transcript variants due to aberrant splicing are found in predicted biallelic *TP53* samples. **A** *TP53* transcript variants translated to isoforms of various types (*α*, *β*, *γ*) and variant lengths (*full length* (TA), Δ40, Δ133, Δ160). **B** Genomic and transcriptomic abnormalities of known and predicted *TP53* samples for NDMM and RRMM. **C** Three predicted biallelic samples with copy number deletion have either high expression *β* or low expression *α*. **D** Retained introns detected in a predicted biallelic sample led to a novel transcript variant with protein-coding potential. TA transactivation, dPSI delta percent spliced in, DBD DNA binding domain, TAD transactivation domain.

Based on established studies [15] and databases [42], we annotated all protein-coding transcript variants of *TP53* listed in the Gencode [43] hg38 V35 genome annotation (Fig. 4A). In cancers, the expression of $\alpha ,\beta \,{\rm{and}}\,\gamma$ isoforms is frequently switched [44]. Inclusion of the cryptic exon(s) between exons 9 and 10 results in a switch to the $\beta \,{\rm{and}}\,\gamma$ isoforms (Fig. 4A). Among all predicted biallelic samples, we observed several that underwent dramatic splicing changes of the cryptic exon (Fig. 4B), leading to the dominant expression of $\beta /\gamma$ over $\alpha$. For instance, inclusion of the cryptic exon (*dPSI* = −0.54, versus normal) was observed in sample MMRF_1922 (Fig. 4C), indicating the dominant expression of $\beta /\gamma$ transcripts over $\alpha$ (Fig. 4B). We further measured the expression levels of different transcript variants (*α*, *β* and *γ*) and transcript variants with different lengths (full length (TA), ∆40, ∆133 and ∆160) (Supplementary Fig. 4A-C). We confirmed that aberrant inclusion of the cryptic exon resulted in the predominant expression of ${\rm{TAp}}53\beta$, which further led to the imbalanced expression of ${\rm{TAp}}53\beta /\gamma$ over ${\rm{TAp}}53\alpha$. Such an imbalance was reported as a major predictor of poor prognosis in MM [15]. Given that MMRF_1922 had copy number-neutral LOH and a high level of imbalanced expression of ${\rm{TAp}}53\beta /\gamma$ over ${\rm{TAp}}53\alpha$ (ratio = 1.95), it could be reasonably speculated that both alleles underwent cryptic exon inclusion, which further led to dominant expression of high-risk transcripts, which was equivalent to complete inactivation of *TP53*.

Similarly, a few predicted biallelic samples with copy number loss also underwent aberrant cryptic exon inclusion. MMRF_1085_3 had copy number loss (CNV = −0.9) on one allele and cryptic exon inclusion on the other allele (*dPSI* = −0.29, vs. normal, Fig. 4B, C). Some other predicted biallelic samples had a mutation or copy number loss while having a very low expression of all *TP53* transcripts. For instance, MMRF_1450, which was a predicted biallelic sample with copy number loss, had low *TP53* total gene expression (Log_2_(TPM + 1) = 1.01, log_2_FC = −0.89, vs. normal) as well as low $\alpha$ transcript expression (Log_2_(TPM + 1) = 0.41, log_2_FC = −1.59, vs. normal, Fig. 4B, C). This indicated that biallelic *TP53* inactivation could also be defined by copy number loss on one allele and insufficient expression on the remaining allele.

Novel aberrant splicing sites other than the cryptic exon were also observed in predicted biallelic samples. For instance, in sample MMRF_2816 with copy number loss, we observed two novel retained introns between exons 6 and 8 (Fig. 4D). Since the intron retention event was novel, a novel transcript variant assembly and subsequent functional analysis were conducted. Three novel transcript variants with coding potential were inferred (Fig. 4D). Among them, ‘*TP53*-v4’ was predicted to have direct readthrough of exons 6–8, and the resulting protein would terminate within intron 6, resulting in a truncated DNA-binding domain and loss of the tetramerization domain (Fig. 4D and Supplementary Fig. 4D) and protein function [45]. We found that this transcript was expressed in MMRF_2816 (Log_2_(TPM + 1) = 0.92) and minimally expressed in normal BMPCs (Supplementary Fig. 4E).

### Splice site mutations result in aberrant intron splicing

**Fig. 5:**
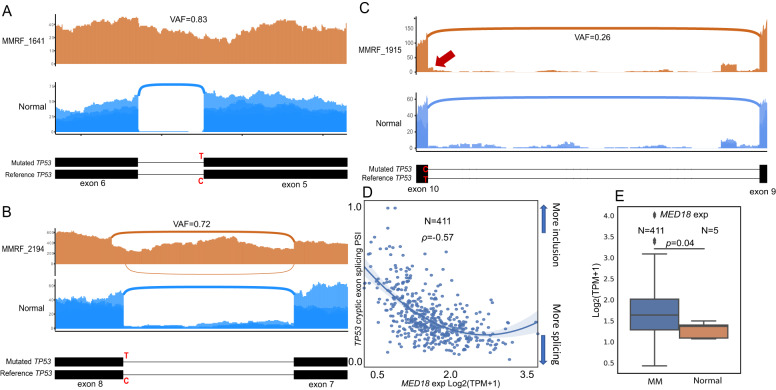
Splice site mutations led to aberrant intron splicing. **A** A 5’ splice site mutation led to retained intron 9. **B** 3’ splice site mutation led to retained intron 10. **C** 3’ splice site mutation led to alternative 3’ splice site splicing. **D** Expression of *MED18* is strongly associated with cryptic exon splicing. **E** Ratio differences between expressed TA normal/abnormal in predicted biallelic samples and normal BMPCs.

After identifying aberrant splicing as a possible source that contributes to biallelic inactivation, we next tried to identify the reasons that lead to such abnormality. Previous research indicated that splice site mutations could lead to alternative splicing [46]. We subsequently examined samples with splice site mutations in the population. Out of six samples with *TP53* splice site mutations, three were found to have aberrant splicing (Fig. 5A–C), while the other three were not due to low VAF of the splice site mutations (average VAF = 0.1), and VAF was positively correlated with aberrant splicing levels (Supplementary Fig. 5A–C).

A C > T substitution (rs1131691042, g.7675052C>T) was found at the 3’ splice site of exon 5 in MMRF_1641 (Fig. 5A). Another C > T substitution (rs1555525367, g.7673838C>T) was found at the 5’ splice site of exon 8 in MMRF_2194 (Fig. 5B). Both single nucleotide variations had high variant allele frequencies (VAF = 0.83 and 0.72), leading to aberrant intron retention (Fig. 5A, B). A T > C substitution (rs1555526335, g.7675235T>C, VAF = 0.26) was found at the 5’ end of exon 10 in MMRF_1915, resulting in a novel alternative 3’ splice site event (Fig. 5C). This novel splice site shared a similar splicing pattern with the previously reported TP53$\Psi$, of which the aberrant splice site was on the 3’ between exon 6 and 7 [47].

### Additional mechanisms controlling cryptic splicing of *TP53*

Interestingly, the inclusion of the cryptic exons described above was not related to splice site mutations and was likely governed by various factors, such as splicing factors [48] and miRNAs [49], each of which may be involved in regulating the exclusion/inclusion of exons and introns. Most likely, the inclusion of cryptic exons is regulated by the combinational effects of these factors. To potentially identify a master regulator that further controls exon inclusion in MM, we conducted a correlation analysis between gene expression and cryptic exon splicing levels. *MED18* expression had the highest correlation (Fig. 5D) with dPSI in *TP53* cryptic splicing ($\rho =-0.57$, Spearman correlation). Among 411 MM samples with more retained cryptic exons (dPSI < 0), *MED18* expression was significantly higher than that in normal BMPCs (FC = 1.3, *p* = 0.04, Mann‒Whitney *U* test, Fig. 5E). *MED18* encodes a component of the mediator complex that binds to DNA, activating transcription via RNA polymerase II (RNAPII) [50], of which the carboxy-terminal domain (CTD) regulates exon in-/exclusion via transcription elongation [51]. Inhibition of RNAPII elongation has been shown to result in more exon inclusion in vitro and vice versa [52]. Our observation suggested that the downregulation of *MED18* may lead to more cryptic exon inclusion, possibly via the downregulation of RNAPII activity.

## Discussion

Historically, deletion of the short arm of chromosome 17p, as detected by cytogenetics and FISH, has been associated with poor outcomes in MM, and the gene of interest on 17p is *TP53* [7, 53]. As technologies have evolved, we have learned that deletion of *TP53* alone may not be associated with poor outcomes. Instead, biallelic inactivation of both copies, through deletion or mutation, is truly associated with poor outcome, and deletion alone is not [9]. This situation has been identified not only in MM but also in myelodysplastic syndromes, myelofibrosis, and acute myeloid leukemia [12].

Given that biallelic inactivation of genes can arise not only through deletion and mutation but also by a variety of other means, it stands to reason that there may be additional patients with biallelic inactivation of *TP53* who are not identified with current DNA tests. However, the downstream signature of biallelic inactivation may be detectable through expression profiling, which can give a more complete picture of the cellular response.

In this study, we demonstrated that biallelic *TP53* samples in MM could be accurately predicted by the transcriptomic signature. This signature predicted biallelic samples from newly diagnosed and relapsed populations with consistently high accuracy. As a result, 26 newly diagnosed and 5 relapsed samples with confirmed monoallelic *TP53* status were predicted as biallelic *TP53* patients. Their survival showed no significant difference from known biallelic patients but was significantly worse than that of confirmed monoallelic or wild-type patients. This is in line with previous reports in which patients with monoallelic *TP53* copy number loss or mutation were also associated with inferior survival [54], which could be partly accounted for by an underestimation of the population of biallelic inactivation.

From the predicted subgroup, we identified the enrichment of samples with overexpressed high-risk *TP53* transcript variants, which is in line with previous reports [15] and may work in concert with existing monoallelic abnormalities such as copy number loss, resulting in loss of function of both alleles of *TP53*. Such high-risk *TP53* transcript variants were derived from aberrant exon inclusion or intron retention. Moreover, different mechanisms of biallelic inactivation were observed. For instance, low expression of *TP53* was found in a few predicted biallelic samples with existing copy number loss, even though the reason for such low expression requires further investigation. These samples could be potentially biallelic, while the second hit might not be detected due to various reasons, including DNA methylation of the *TP53* promoter, cryptic rearrangements that are difficult to resolve with short-read sequencing, or germline variants that are masked by somatic analysis. However, the second hit was unlikely to be caused by germline *TP53* pathogenic mutations due to its ultra-low frequency (<0.2%) in the CoMMpass MM population [55].

Previous studies indicated that splice site mutations in *TP53* resulted in aberrant splicing in colorectal cancer [46]. Here, we confirmed that such mutations not only led to aberrant splicing but also generated high-risk transcript variants, some of which were not previously documented. This indicated that even though *TP53* has been extensively studied, the complete *TP53* landscape is highly heterogeneous among MM patients, illustrating a need for further investigation. Moreover, aberrant splicing may work in concert with genomic variations to cause biallelic status, indicating a future need to combine genomic and transcriptomic features to confirm the biallelic status of *TP53* in the clinical setting.

*TP53* splicing is a complex process and possibly regulated by multiple factors simultaneously [48]. Most likely, the splicing level is determined by a combination of factors. Nonetheless, we demonstrated that *MED18* may serve as a master regulator to control the cryptic exon splicing level of *TP53*. Numerous reports have suggested regulatory roles of mediator complex members in RNA splicing [56]. The strong correlation between *MED18* expression and cryptic exon splicing levels indicated that by targeting the mediator complex, the ‘hazardous’ *TP53* isoforms could potentially be turned into tumor-suppressing isoforms, which may offer an alternative approach for targeting aberrant *TP53* in MM. However, as an essential component in the mediator complex, *MED18* most likely controls numerous splicing events other than *TP53* cryptic exons.

### Supplementary information

supplementary material

## Data Availability

All sequencing datasets have been previously published and can be accessed through dbGAP Study Accession number phs000748 (MMRF CoMMpass dataset) and GEO GSE110486 (normal plasma cell dataset).

## References

[1] Chng WJ, Dispenzieri A, Chim CS, Fonseca R, Goldschmidt H, Lentzsch S (2014). IMWG consensus on risk stratification in multiple myeloma. Leukemia.

[2] Palumbo A, Avet-Loiseau H, Oliva S, Lokhorst HM, Goldschmidt H, Rosinol L (2015). Revised international staging system for multiple myeloma: a report from international myeloma working group. J Clin Oncol.

[3] Caers J, Garderet L, Kortum KM, O’Dwyer ME, van de Donk N, Binder M (2018). European Myeloma Network recommendations on tools for the diagnosis and monitoring of multiple myeloma: what to use and when. Haematologica.

[4] D’Agostino M, Cairns DA, Lahuerta JJ, Wester R, Bertsch U, Waage A (2022). Second Revision of the International Staging System (R2-ISS) for Overall Survival in Multiple Myeloma: a European Myeloma Network (EMN) Report Within the HARMONY Project. J Clin Oncol.

[5] Drach J, Ackermann J, Fritz E, Kromer E, Schuster R, Gisslinger H (1998). Presence of a p53 gene deletion in patients with multiple myeloma predicts for short survival after conventional-dose chemotherapy. Blood.

[6] Walker BA, Leone PE, Chiecchio L, Dickens NJ, Jenner MW, Boyd KD (2010). A compendium of myeloma-associated chromosomal copy number abnormalities and their prognostic value. Blood.

[7] Thanendrarajan S, Tian E, Qu P, Mathur P, Schinke C, van Rhee F (2017). The level of deletion 17p and bi-allelic inactivation of TP53 has a significant impact on clinical outcome in multiple myeloma. Haematologica.

[8] Thakurta A, Ortiz M, Blecua P, Towfic F, Corre J, Serbina NV (2019). High subclonal fraction of 17p deletion is associated with poor prognosis in multiple myeloma. Blood.

[9] Walker BA, Mavrommatis K, Wardell CP, Ashby TC, Bauer M, Davies F (2019). A high-risk, double-hit, group of newly diagnosed myeloma identified by genomic analysis. Leukemia.

[10] Boyle EM, Deshpande S, Tytarenko R, Ashby C, Wang Y, Bauer MA (2021). The molecular make up of smoldering myeloma highlights the evolutionary pathways leading to multiple myeloma. Nat Commun.

[11] Weinhold N, Ashby C, Rasche L, Chavan SS, Stein C, Stephens OW (2016). Clonal selection and double-hit events involving tumor suppressor genes underlie relapse in myeloma. Blood.

[12] Bernard E, Nannya Y, Hasserjian RP, Devlin SM, Tuechler H, Medina-Martinez JS (2020). Implications of TP53 allelic state for genome stability, clinical presentation and outcomes in myelodysplastic syndromes. Nat Med.

[13] Gagelmann N, Badbaran A, Salit RB, Schroeder T, Gurnari C, Pagliuca S (2023). Impact of TP53 on outcome of patients with myelofibrosis undergoing hematopoietic stem cell transplantation. Blood.

[14] Teoh PJ, Chung TH, Sebastian S, Choo SN, Yan J, Ng SB (2014). p53 haploinsufficiency and functional abnormalities in multiple myeloma. Leukemia.

[15] Rojas EA, Corchete LA, De Ramon C, Krzeminski P, Quwaider D, Garcia-Sanz R (2022). Expression of p53 protein isoforms predicts survival in patients with multiple myeloma. Am J Hematol.

[16] Cibulskis K, Lawrence MS, Carter SL, Sivachenko A, Jaffe D, Sougnez C (2013). Sensitive detection of somatic point mutations in impure and heterogeneous cancer samples. Nat biotechnology.

[17] Kim S, Scheffler K, Halpern AL, Bekritsky MA, Noh E, Källberg M (2018). Strelka2: fast and accurate calling of germline and somatic variants. Nat Methods.

[18] Cooke DP, Wedge DC, Lunter G (2021). A unified haplotype-based method for accurate and comprehensive variant calling. Nat Biotechnol.

[19] Narzisi G, Corvelo A, Arora K, Bergmann EA, Shah M, Musunuri R (2018). Genome-wide somatic variant calling using localized colored de Bruijn graphs. Commun Biol.

[20] Van der Auwera GA, Carneiro MO, Hartl C, Poplin R, Del Angel G, Levy‐Moonshine A (2013). From FastQ data to high‐confidence variant calls: the genome analysis toolkit best practices pipeline. Curr Protoc Bioinforma.

[21] Wagner GP, Kin K, Lynch VJ (2012). Measurement of mRNA abundance using RNA-seq data: RPKM measure is inconsistent among samples. Theory Biosci.

[22] Patro R, Duggal G, Love MI, Irizarry RA, Kingsford C (2017). Salmon provides fast and bias-aware quantification of transcript expression. Nat methods.

[23] Ritchie ME, Phipson B, Wu DI, Hu Y, Law CW, Shi W (2015). limma powers differential expression analyses for RNA-sequencing and microarray studies. Nucleic acids Res.

[24] Zhan F, Huang Y, Colla S, Stewart JP, Hanamura I, Gupta S (2006). The molecular classification of multiple myeloma. Blood.

[25] Shao M, Kingsford C (2017). Accurate assembly of transcripts through phase-preserving graph decomposition. Nat Biotechnol.

[26] Gasteiger E, Gattiker A, Hoogland C, Ivanyi I, Appel RD, Bairoch A (2003). ExPASy: the proteomics server for in-depth protein knowledge and analysis. Nucleic acids Res.

[27] Lu S, Wang J, Chitsaz F, Derbyshire MK, Geer RC, Gonzales NR (2020). CDD/SPARCLE: the conserved domain database in 2020. Nucleic Acids Res.

[28] Wang J, Vasaikar S, Shi Z, Greer M, Zhang B (2017). WebGestalt 2017: a more comprehensive, powerful, flexible and interactive gene set enrichment analysis toolkit. Nucleic acids Res.

[29] Subramanian A, Tamayo P, Mootha VK, Mukherjee S, Ebert BL, Gillette MA (2005). Gene set enrichment analysis: a knowledge-based approach for interpreting genome-wide expression profiles. Proc Natl Acad Sci USA.

[30] Kanehisa M, Goto S (2000). KEGG: Kyoto encyclopedia of genes and genomes. Nucleic Acids Res.

[31] Slenter DN, Kutmon M, Hanspers K, Riutta A, Windsor J, Nunes N (2018). WikiPathways: a multifaceted pathway database bridging metabolomics to other omics research. Nucleic Acids Res.

[32] Hänzelmann S, Castelo R, Guinney J (2013). GSVA: gene set variation analysis for microarray and RNA-seq data. BMC Bioinforma.

[33] Pedregosa F, Varoquaux G, Gramfort A, Michel V, Thirion B, Grisel O (2011). Scikit-learn: Machine learning in Python. J Mach Learn Res.

[34] Shen S, Park JW, Lu Z-X, Lin L, Henry MD, Wu YN (2014). rMATS: robust and flexible detection of differential alternative splicing from replicate RNA-Seq data. Proc Natl Acad Sci USA.

[35] Saito T, Rehmsmeier M (2015). The precision-recall plot is more informative than the ROC plot when evaluating binary classifiers on imbalanced datasets. PLoS One.

[36] Jeni LA, Cohn JF, De La Torre F, editors. Facing imbalanced data--recommendations for the use of performance metrics. (IEEE, 2013). 10.1109/ACII.2013.47 PMC4285355 25574450

[37] Qu Y, Li J, Cai Q, Liu B (2014). Hec1/Ndc80 is overexpressed in human gastric cancer and regulates cell growth. J Gastroenterol.

[38] Wu P, Walker BA, Brewer D, Gregory WM, Ashcroft J, Ross FM (2011). A gene expression–based predictor for myeloma patients at high risk of developing bone disease on bisphosphonate treatment. Clin Cancer Res.

[39] Mason MJ, Schinke C, Eng CLP, Towfic F, Gruber F, Dervan A (2020). Multiple Myeloma DREAM Challenge reveals epigenetic regulator PHF19 as marker of aggressive disease. Leukemia.

[40] Chen J (2016). The cell-cycle arrest and apoptotic functions of p53 in tumor initiation and progression. Cold Spring Harb Perspect Med.

[41] Anbarasan T, Bourdon J-C (2019). The emerging landscape of p53 isoforms in physiology, cancer and degenerative diseases. Int J Mol Sci.

[42] Zhou X, Edmonson MN, Wilkinson MR, Patel A, Wu G, Liu Y (2016). Exploring genomic alteration in pediatric cancer using ProteinPaint. Nat Genet.

[43] Frankish A, Diekhans M, Ferreira A-M, Johnson R, Jungreis I, Loveland J (2019). GENCODE reference annotation for the human and mouse genomes. Nucleic acids Res.

[44] Bourdon JC (2014). p53 isoforms change p53 paradigm. Mol Cell Oncol.

[45] Baugh EH, Ke H, Levine AJ, Bonneau RA, Chan CS (2018). Why are there hotspot mutations in the TP53 gene in human cancers?. Cell Death Differ.

[46] Smeby J, Sveen A, Eilertsen IA, Danielsen SA, Hoff AM, Eide PW (2019). Transcriptional and functional consequences of TP53 splice mutations in colorectal cancer. Oncogenesis.

[47] Senturk S, Yao Z, Camiolo M, Stiles B, Rathod T, Walsh AM (2014). p53Ψ is a transcriptionally inactive p53 isoform able to reprogram cells toward a metastatic-like state. Proc Natl Acad Sci USA.

[48] Kędzierska H, Piekiełko-Witkowska A (2017). Splicing factors of SR and hnRNP families as regulators of apoptosis in cancer. Cancer Lett.

[49] Jones MF, Lal A (2012). MicroRNAs, wild-type and mutant p53: more questions than answers. RNA Biol.

[50] Sato S, Tomomori-Sato C, Banks CAS, Sorokina I, Parmely TJ, Kong SE (2003). Identification of Mammalian Mediator Subunits with Similarities to Yeast Mediator Subunits Srb5, Srb6, Med11, and Rox3* 210. J Biol Chem.

[51] Muñoz MJ, Santangelo MSP, Paronetto MP, de la Mata M, Pelisch F, Boireau S (2009). DNA damage regulates alternative splicing through inhibition of RNA polymerase II elongation. Cell.

[52] Ip JY, Schmidt D, Pan Q, Ramani AK, Fraser AG, Odom DT (2011). Global impact of RNA polymerase II elongation inhibition on alternative splicing regulation. Genome Res.

[53] Boyd KD, Ross FM, Tapper WJ, Chiecchio L, Dagrada G, Konn ZJ (2011). The clinical impact and molecular biology of del (17p) in multiple myeloma treated with conventional or thalidomide‐based therapy. Genes Chromosomes Cancer.

[54] Corre J, Perrot A, Caillot D, Belhadj K, Hulin C, Leleu X (2021). del (17p) without TP53 mutation confers a poor prognosis in intensively treated newly diagnosed patients with multiple myeloma. Blood.

[55] Thibaud S, Etra A, Subaran R, Soens Z, Newman S, Chen R (2021). Pathogenic germline variants in multiple myeloma. Blood.

[56] Huang Y, Li W, Yao X, Lin Q-J, Yin J-W, Liang Y (2012). Mediator complex regulates alternative mRNA processing via the MED23 subunit. Mol Cell.

