# Supplementary material for: Identifying novel mechanisms of biallelic TP53 loss refines poor outcome for patients with multiple myeloma
Source: Blood Cancer J. 2023 Sep 11;13(1):144. doi: 10.1038/s41408-023-00919-2 (PMC10495448; doi:10.1038/s41408-023-00919-2)
Supplement: Supplementary file 1 — supplementary material [file 41408_2023_919_MOESM1_ESM.pdf]

Title: Identifying novel mechanisms of biallelic *TP53* loss refines poor outcome for patients with multiple myeloma

Authors:

Enze Liu[1], Parvathi Sudha[1], Nathan Becker[1], Oumaima Jaouadi[1], Attaya Suvannasankha[1], Kelvin Lee[1], Rafat Abonour[1], Mohammad Abu Zaid[1], Brian A. Walker[1,2]

Affiliations:

[1]Melvin and Bren Simon Comprehensive Cancer Center, Division of Hematology and Oncology, School of Medicine, Indiana University, Indianapolis, IN, USA

[2]Center for Computational Biology and Bioinformatics, School of Medicine, Indiana University, Indianapolis, IN, USA

Correspondence: Brian A. Walker, C310 Walther Hall, 980 W Walnut St, Indiana University, Indianapolis, IN, 46202. bw75@iu.edu

Supplementary Figures

Supplementary Figure 1

The Predicted model and characteristics of predicted samples: (**A**). Differentially expressed genes between known biallelic *TP53* and *TP53* WT samples. Green: 1.5<FC<2, Yellow: 2<FC<2.5, Orange: 2.5<FC<3, Red>3. (**B**). Selected random forest regression model. (**C**). A PPI network cluster of 14 out of 16 genes used in the random forest model. Blue dots: 14 genes; Grey dots: neighbor genes documented in bioGRID human PPI network. The network was retrieved using webgsealt[1]**.** (**D**). Dysregulated pathways of individual samples from GSVA. (**E**). Supervised clustering of NDMM population using 7 pre-defined gene signatures. CD-1, CD-2, HP, LB, MF, MS and PR were previously defined MM subpopulation that contain various molecular features[2].

(A)

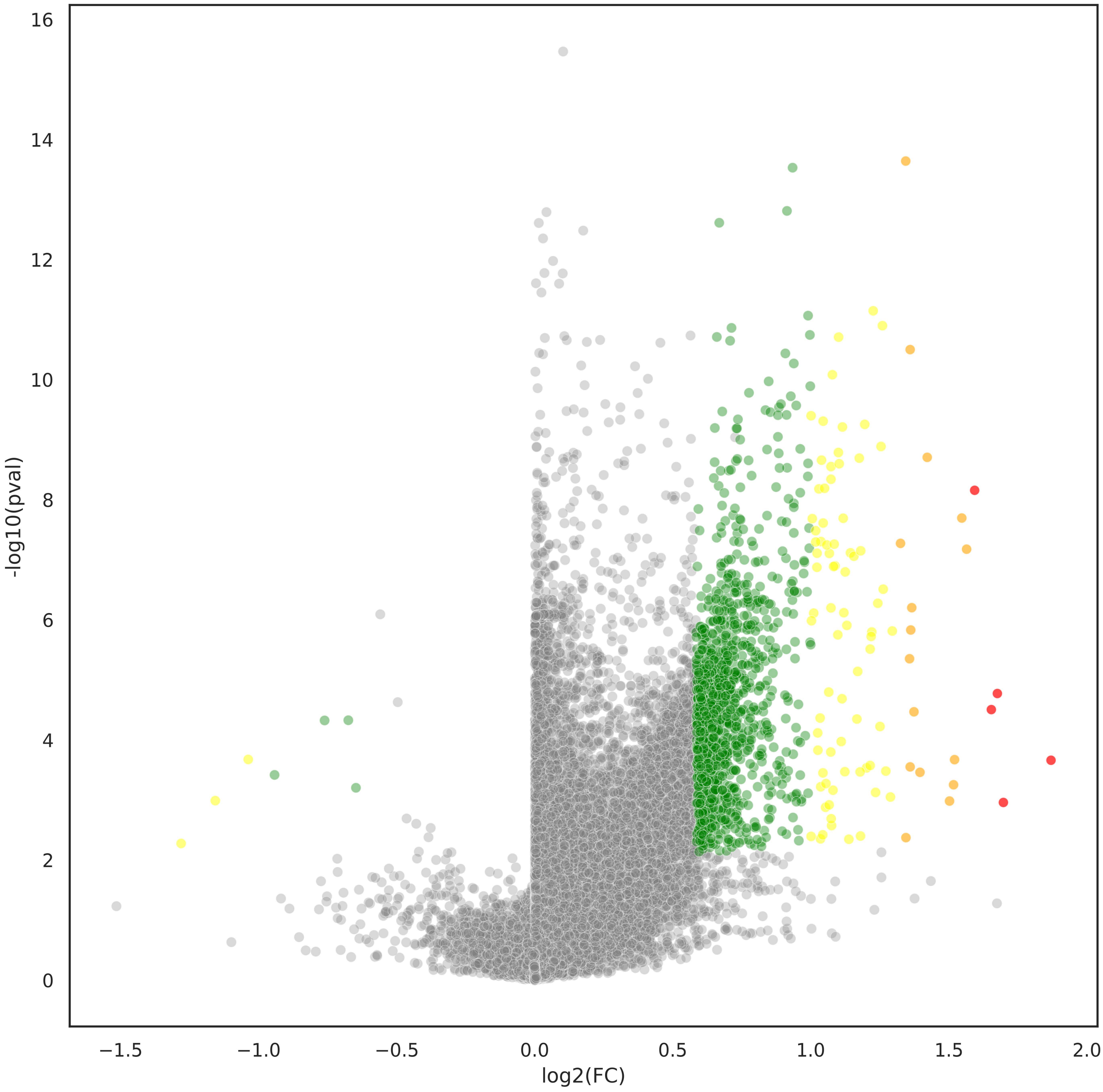

(B)

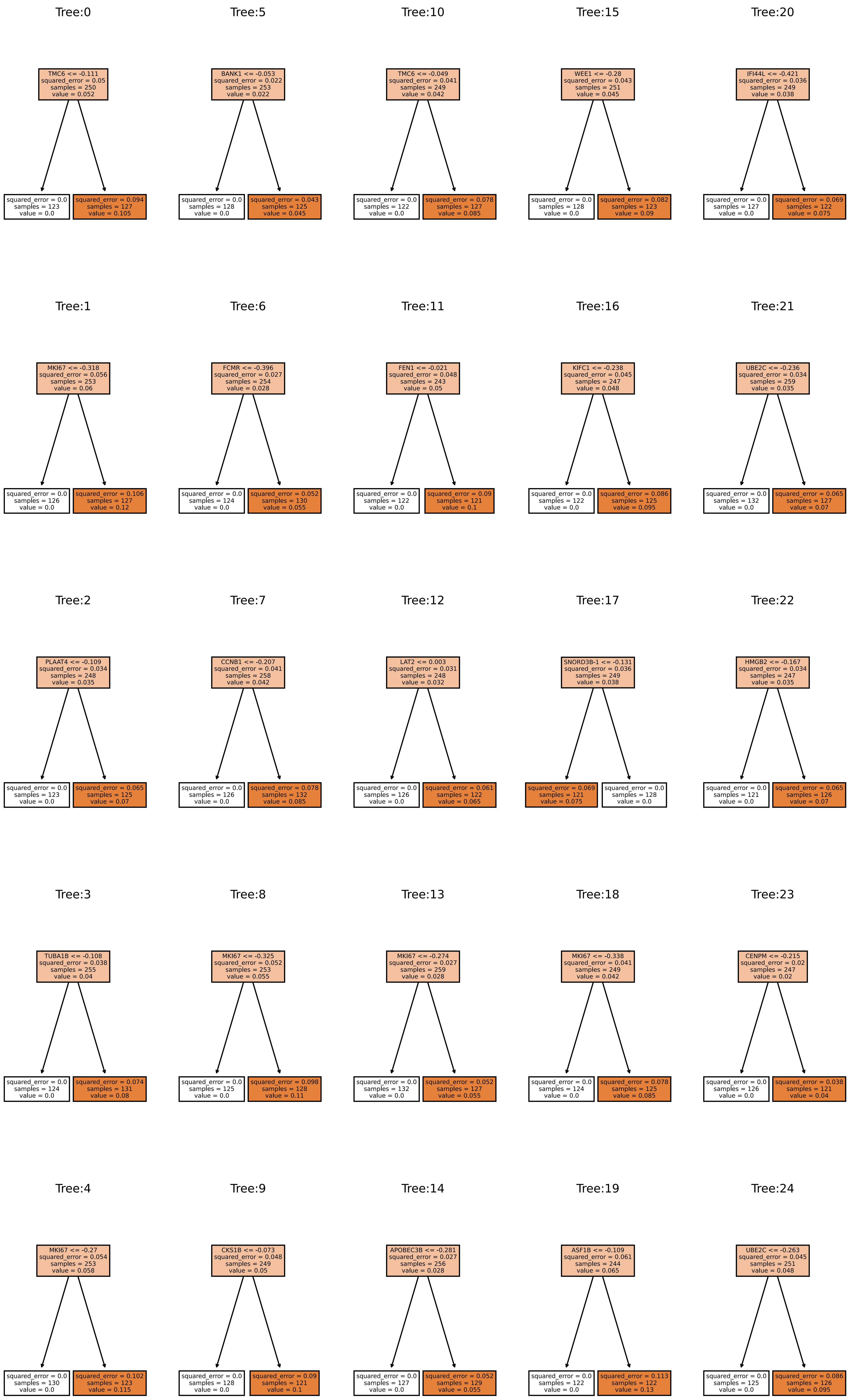

Tree:0
TMC6 <= -0.111
squared_error = 0.05
samples = 250
value = 0.052
squared_error = 0.0
samples = 123
value = 0.0
squared_error = 0.094
samples = 127
value = 0.105
Tree:5
BANK1 <= -0.053
squared_error = 0.022
samples = 253
value = 0.022
squared_error = 0.0
samples = 128
value = 0.0
squared_error = 0.043
samples = 125
value = 0.045
Tree:10
TMC6 <= -0.049
squared_error = 0.041
samples = 249
value = 0.042
squared_error = 0.0
samples = 122
value = 0.0
squared_error = 0.078
samples = 127
value = 0.085
Tree:15
WEE1 <= -0.28
squared_error = 0.043
samples = 251
value = 0.045
squared_error = 0.0
samples = 128
value = 0.0
squared_error = 0.082
samples = 123
value = 0.09
Tree:20
IFI44L <= -0.421
squared_error = 0.036
samples = 249
value = 0.038
squared_error = 0.0
samples = 127
value = 0.0
squared_error = 0.069
samples = 122
value = 0.075
Tree:1
MKI67 <= -0.318
squared_error = 0.056
samples = 253
value = 0.06
squared_error = 0.0
samples = 126
value = 0.0
squared_error = 0.106
samples = 127
value = 0.12
Tree:6
FCMR <= -0.396
squared_error = 0.027
samples = 254
value = 0.028
squared_error = 0.0
samples = 124
value = 0.0
squared_error = 0.052
samples = 130
value = 0.055
Tree:11
FEN1 <= -0.021
squared_error = 0.048
samples = 243
value = 0.05
squared_error = 0.0
samples = 122
value = 0.0
squared_error = 0.09
samples = 121
value = 0.1
Tree:16
KIFC1 <= -0.238
squared_error = 0.045
samples = 247
value = 0.048
squared_error = 0.0
samples = 122
value = 0.0
squared_error = 0.086
samples = 125
value = 0.095
Tree:21
UBE2C <= -0.236
squared_error = 0.034
samples = 259
value = 0.035
squared_error = 0.0
samples = 132
value = 0.0
squared_error = 0.065
samples = 127
value = 0.07
Tree:2
PLAAT4 <= -0.109
squared_error = 0.034
samples = 248
value = 0.035
squared_error = 0.0
samples = 123
value = 0.0
squared_error = 0.065
samples = 125
value = 0.07
Tree:7
CCNB1 <= -0.207
squared_error = 0.041
samples = 258
value = 0.042
squared_error = 0.0
samples = 126
value = 0.0
squared_error = 0.078
samples = 132
value = 0.085
Tree:12
LAT2 <= 0.003
squared_error = 0.031
samples = 248
value = 0.032
squared_error = 0.0
samples = 126
value = 0.0
squared_error = 0.061
samples = 122
value = 0.065
Tree:17
SNORD3B-1 <= -0.131
squared_error = 0.036
samples = 249
value = 0.038
squared_error = 0.069
samples = 121
value = 0.075
squared_error = 0.0
samples = 128
value = 0.0
Tree:22
HMGB2 <= -0.167
squared_error = 0.034
samples = 247
value = 0.035
squared_error = 0.0
samples = 121
value = 0.0
squared_error = 0.065
samples = 126
value = 0.07
Tree:3
TUBA1B <= -0.108
squared_error = 0.038
samples = 255
value = 0.04
squared_error = 0.0
samples = 124
value = 0.0
squared_error = 0.074
samples = 131
value = 0.08
Tree:8
MKI67 <= -0.325
squared_error = 0.052
samples = 253
value = 0.055
squared_error = 0.0
samples = 125
value = 0.0
squared_error = 0.098
samples = 128
value = 0.11
Tree:13
MKI67 <= -0.274
squared_error = 0.027
samples = 259
value = 0.028
squared_error = 0.0
samples = 132
value = 0.0
squared_error = 0.052
samples = 127
value = 0.055
Tree:18
MKI67 <= -0.338
squared_error = 0.041
samples = 249
value = 0.042
squared_error = 0.0
samples = 124
value = 0.0
squared_error = 0.078
samples = 125
value = 0.085
Tree:23
CENPM <= -0.215
squared_error = 0.02
samples = 247
value = 0.02
squared_error = 0.0
samples = 126
value = 0.0
squared_error = 0.038
samples = 121
value = 0.04
Tree:4
MKI67 <= -0.27
squared_error = 0.054
samples = 253
value = 0.058
squared_error = 0.0
samples = 130
value = 0.0
squared_error = 0.102
samples = 123
value = 0.115
Tree:9
CKS1B <= -0.073
squared_error = 0.048
samples = 249
value = 0.05
squared_error = 0.0
samples = 128
value = 0.0
squared_error = 0.09
samples = 121
value = 0.1
Tree:14
APOBEC3B <= -0.281
squared_error = 0.027
samples = 256
value = 0.028
squared_error = 0.0
samples = 127
value = 0.0
squared_error = 0.052
samples = 129
value = 0.055
Tree:19
ASF1B <= -0.109
squared_error = 0.061
samples = 244
value = 0.065
squared_error = 0.0
samples = 122
value = 0.0
squared_error = 0.113
samples = 122
value = 0.13
Tree:24
UBE2C <= -0.263
squared_error = 0.045
samples = 251
value = 0.048
squared_error = 0.0
samples = 125
value = 0.0
squared_error = 0.086
samples = 126
value = 0.095

(C)

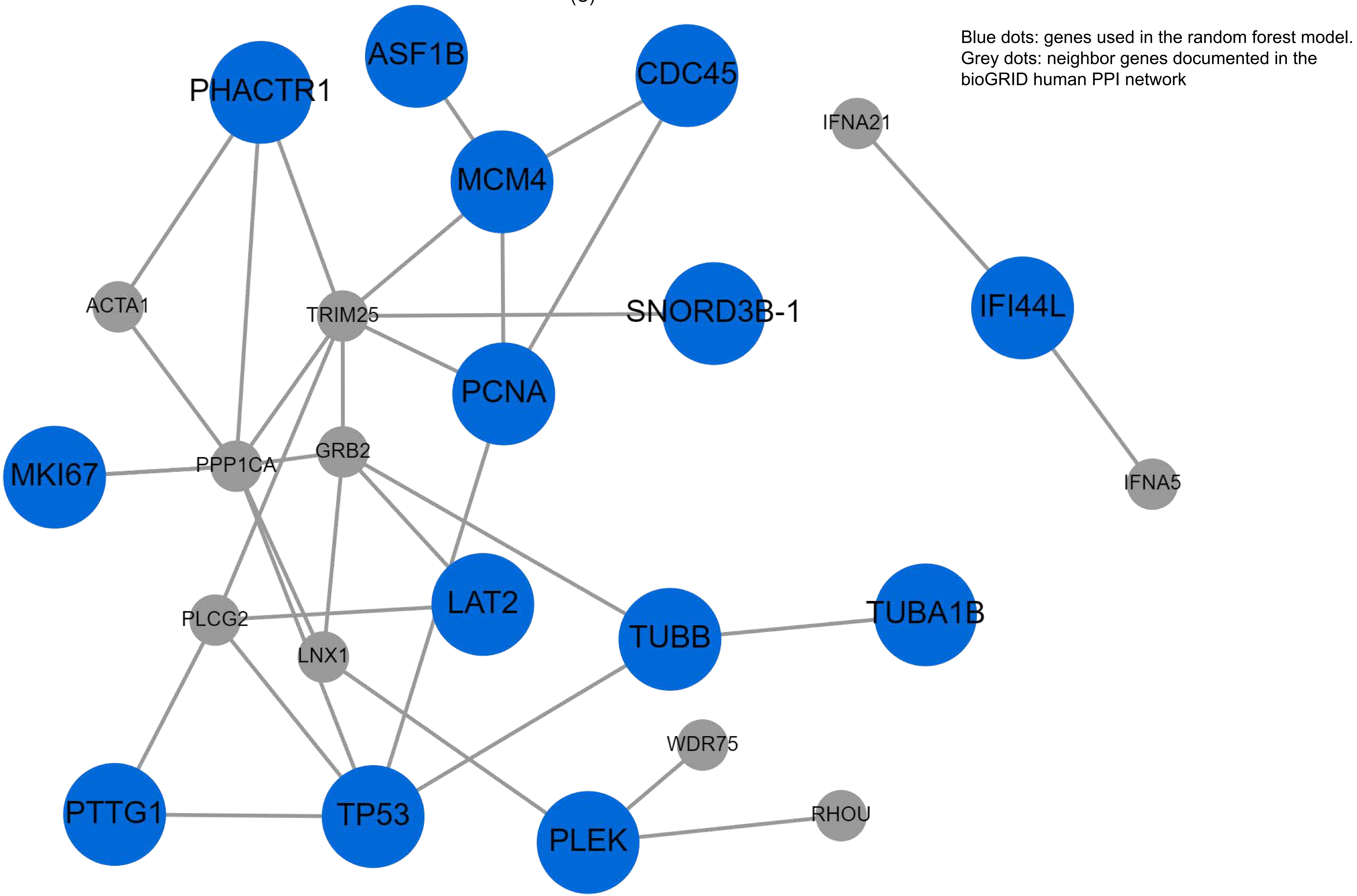
Blue dots: genes used in the random forest model.
Grey dots: neighbor genes documented in the bioGRID human PPI network
PHACTR1
ASF1B
CDC45
MCM4
IFNA21
ACTA1
TRIM25
SNORD3B-1
IFI44L
PCNA
MKI67
PPP1CA
GRB2
IFNA5
LAT2
PLCG2
TUBB
TUBA1B
LNX1
WDR75
RHOU
PTTG1
TP53
PLEK

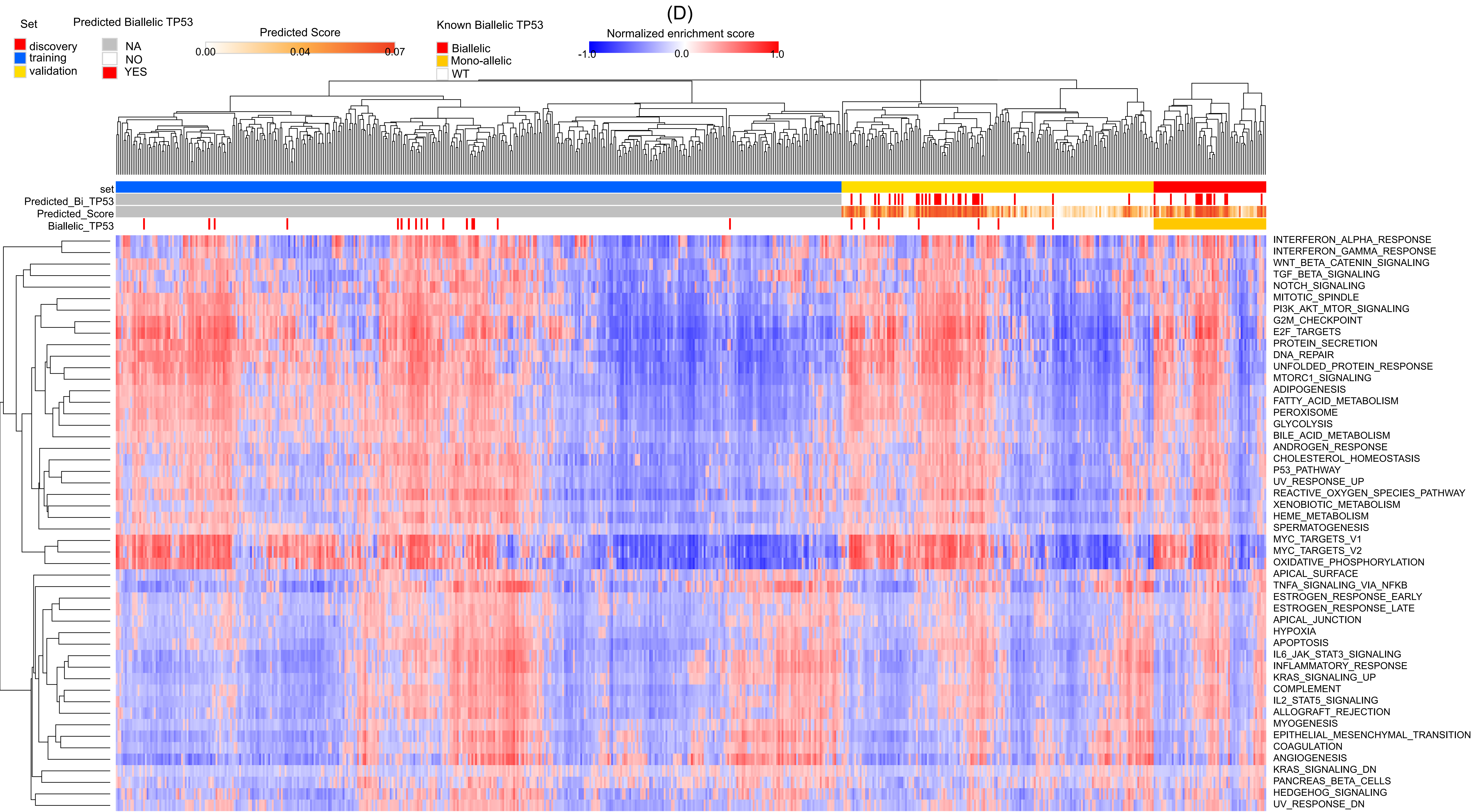

(D)
Set
discovery
training
validation
Predicted Biallelic TP53
NA
NO
YES
Predicted Score
0.00
0.04
0.07
Known Biallelic TP53
Biallelic
Mono-allelic
WT
Normalized enrichment score
-1.0
0.0
1.0
set
Predicted_Bi_TP53
Predicted_Score
Biallelic_TP53
INTERFERON_ALPHA_RESPONSE
INTERFERON_GAMMA_RESPONSE
WNT_BETA_CATENIN_SIGNALING
TGF_BETA_SIGNALING
NOTCH_SIGNALING
MITOTIC_SPINDLE
PI3K_AKT_MTOR_SIGNALING
G2M_CHECKPOINT
E2F_TARGETS
PROTEIN_SECRETION
DNA_REPAIR
UNFOLDED_PROTEIN_RESPONSE
MTORC1_SIGNALING
ADIPOGENESIS
FATTY_ACID_METABOLISM
PEROXISOME
GLYCOLYSIS
BILE_ACID_METABOLISM
ANDROGEN_RESPONSE
CHOLESTEROL_HOMEOSTASIS
P53_PATHWAY
UV_RESPONSE_UP
REACTIVE_OXYGEN_SPECIES_PATHWAY
XENOBIOTIC_METABOLISM
HEME_METABOLISM
SPERMATOGENESIS
MYC_TARGETS_V1
MYC_TARGETS_V2
OXIDATIVE_PHOSPHORYLATION
APICAL_SURFACE
TNFA_SIGNALING_VIA_NFKB
ESTROGEN_RESPONSE_EARLY
ESTROGEN_RESPONSE_LATE
APICAL_JUNCTION
HYPOXIA
APOPTOSIS
IL6_JAK_STAT3_SIGNALING
INFLAMMATORY_RESPONSE
KRAS_SIGNALING_UP
COMPLEMENT
IL2_STAT5_SIGNALING
ALLOGRAFT_REJECTION
MYOGENESIS
EPITHELIAL_MESENCHYMAL_TRANSITION
COAGULATION
ANGIOGENESIS
KRAS_SIGNALING_DN
PANCREAS_BETA_CELLS
HEDGEHOG_SIGNALING
UV_RESPONSE_DN

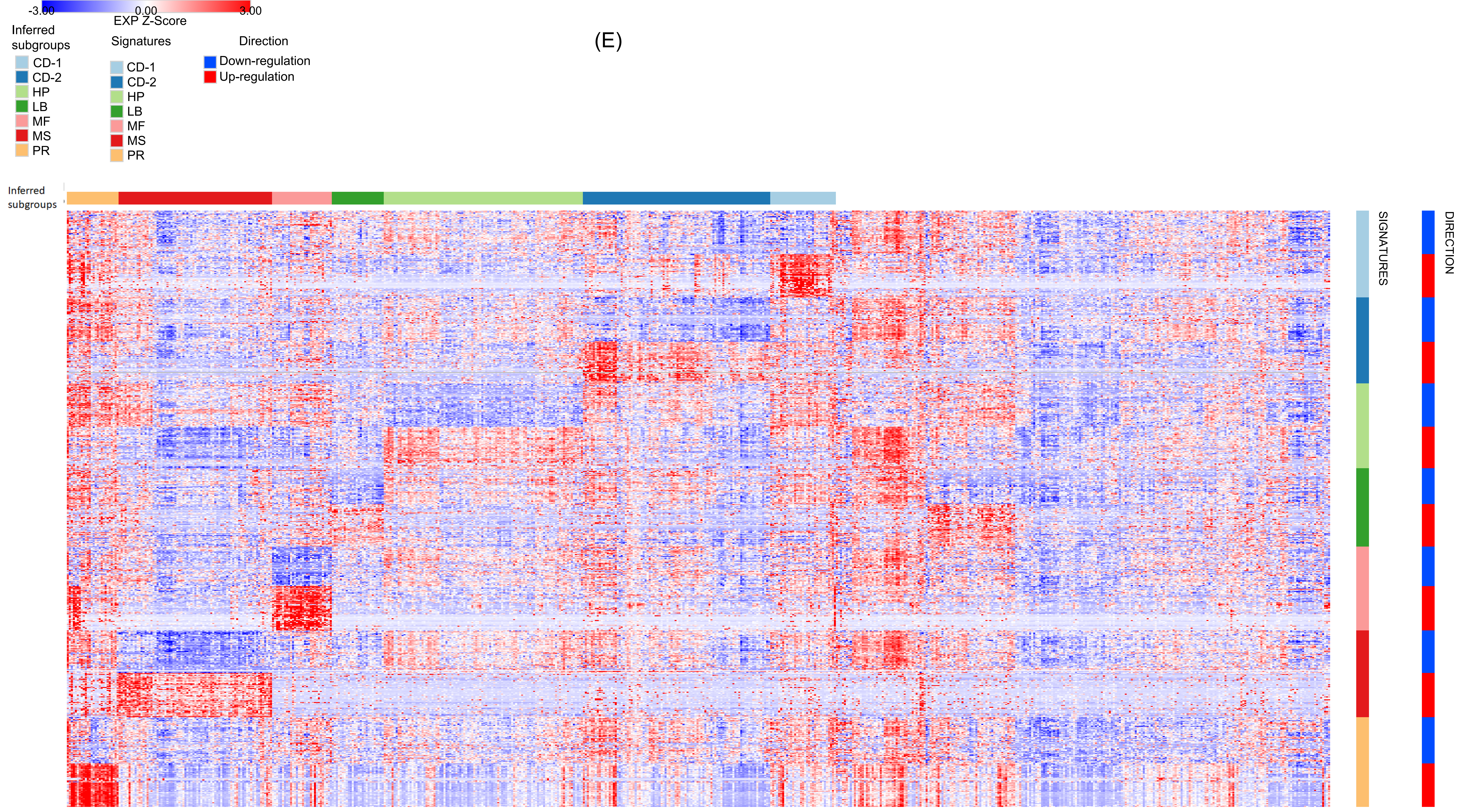

-3.00
0.00
3.00
EXP Z-Score
(E)
Inferred subgroups
CD-1
CD-2
HP
LB
MF
MS
PR
Signatures
CD-1
CD-2
HP
LB
MF
MS
PR
Direction
Down-regulation
Up-regulation
Inferred subgroups
SIGNATURES
DIRECTION

Supplementary Figure 2

(**A**). A bubble plot of significantly dysregulated pathways between 26 predicted biallelic and 36 predicted mono-allelic samples in NDMM cohort (**B**). A bubble plot of significantly dysregulated pathways between 23 known biallelic and 36 predicted mono-allelic samples in NDMM cohort. (**C**). Number of structural variation events in 23 known biallelic, 26 predicted biallelic, 36 predicted monoallelic and 549 WT samples. (**D-E**). Kaplan-Meier curves of progression free survival (PFS) and overall survival (OS) for 23 known biallelic, 62 known monoallelic and 549 WT samples. (**F-G**). Kaplan-Meier curves of PFS and OS for 49 total biallelic (23 known + 26 predicted), remaining 36 monoallelic and 549 WT samples.

(A)

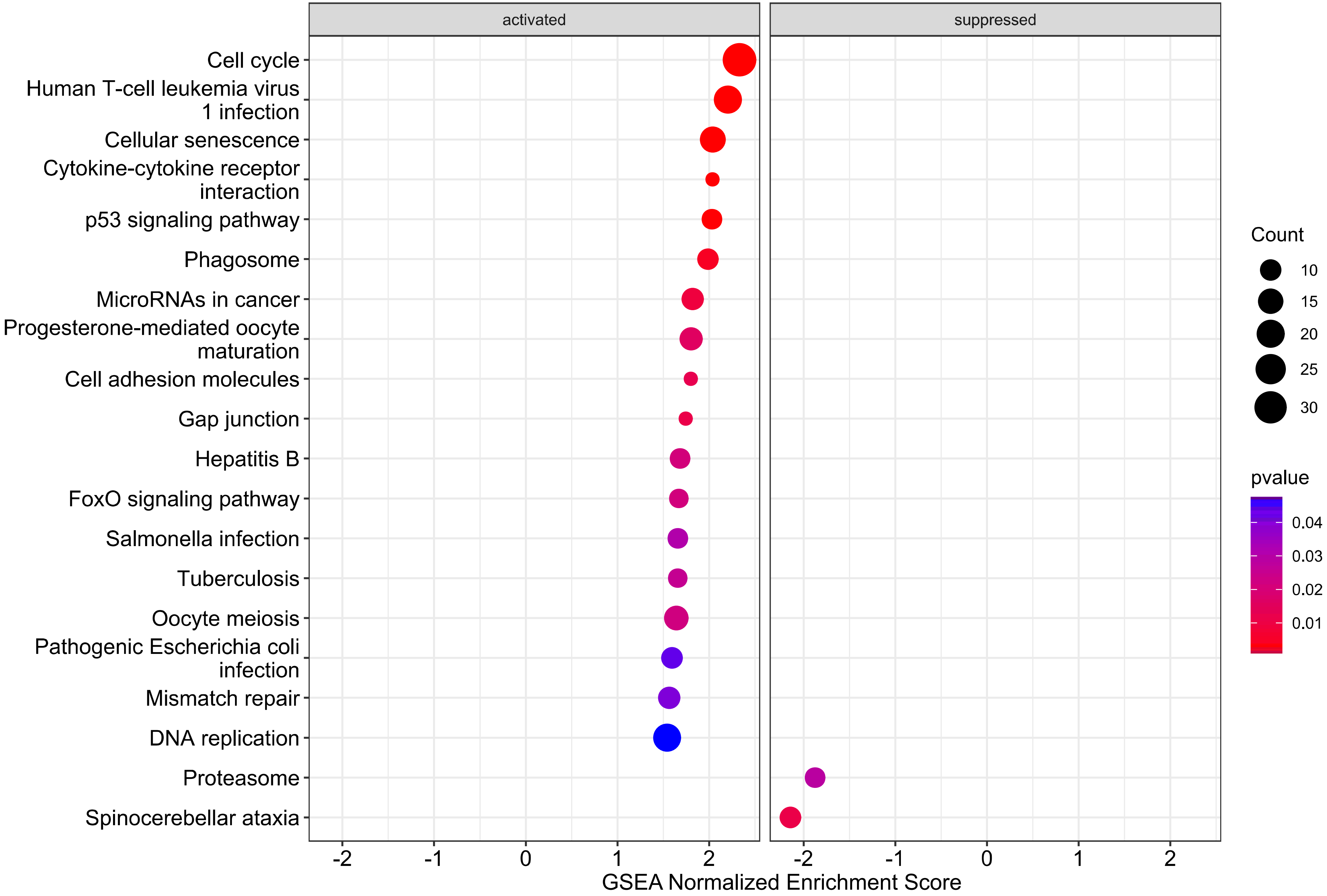

activated
suppressed
Cell cycle
Human T-cell leukemia virus 1 infection
Cellular senescence
Cytokine-cytokine receptor interaction
p53 signaling pathway
Phagosome
MicroRNAs in cancer
Progesterone-mediated oocyte maturation
Cell adhesion molecules
Gap junction
Hepatitis B
FoxO signaling pathway
Salmonella infection
Tuberculosis
Oocyte meiosis
Pathogenic Escherichia coli infection
Mismatch repair
DNA replication
Proteasome
Spinocerebellar ataxia
-2
-1
0
1
2
GSEA Normalized Enrichment Score
Count
10
15
20
25
30
pvalue
0.04
0.03
0.02
0.01

(B)

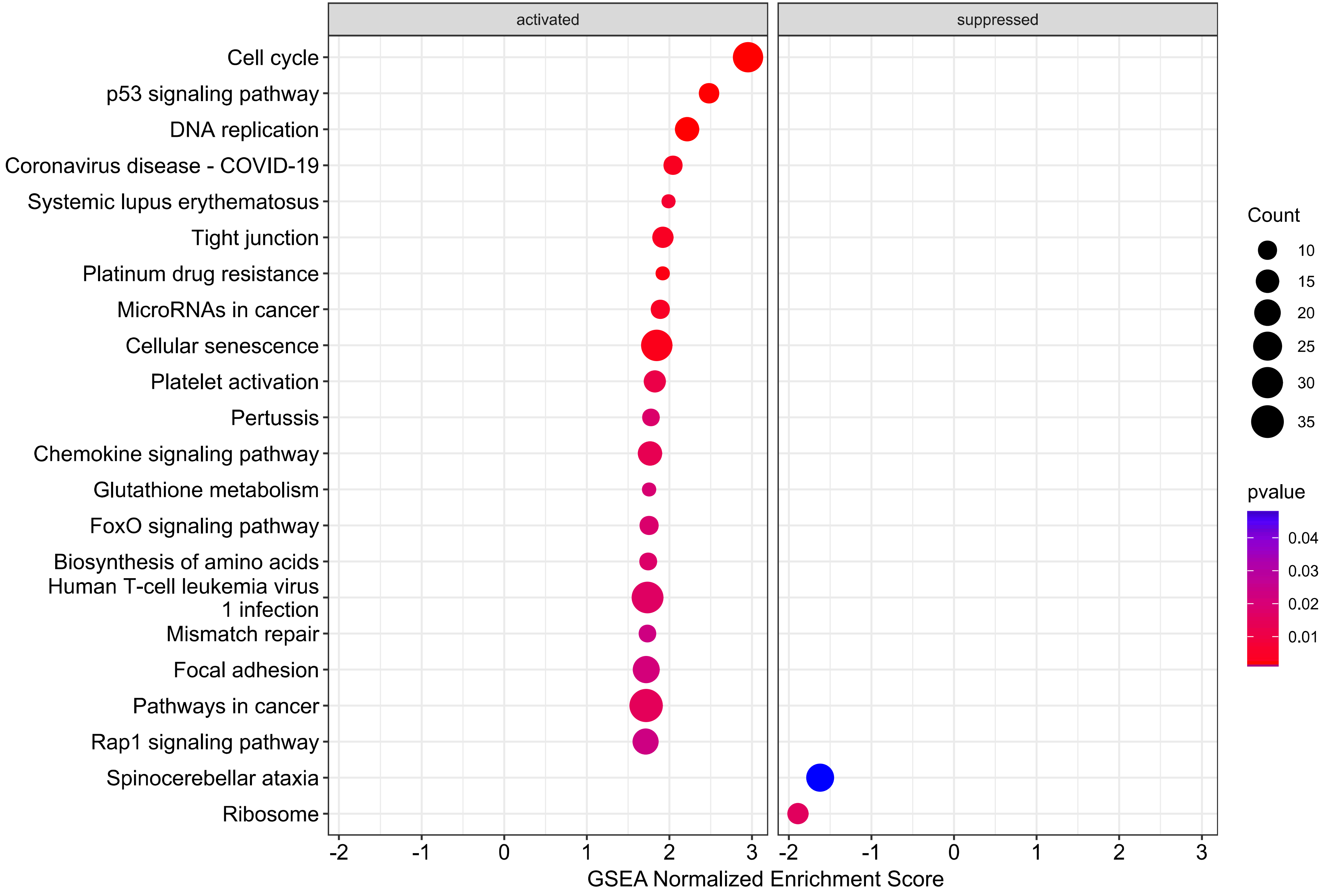

activated
suppressed
Cell cycle
p53 signaling pathway
DNA replication
Coronavirus disease - COVID-19
Systemic lupus erythematosus
Tight junction
Platinum drug resistance
MicroRNAs in cancer
Cellular senescence
Platelet activation
Pertussis
Chemokine signaling pathway
Glutathione metabolism
FoxO signaling pathway
Biosynthesis of amino acids
Human T-cell leukemia virus 1 infection
Mismatch repair
Focal adhesion
Pathways in cancer
Rap1 signaling pathway
Spinocerebellar ataxia
Ribosome
-2 -1 0 1 2 3
-2 -1 0 1 2 3
GSEA Normalized Enrichment Score
Count
10
15
20
25
30
35
pvalue
0.04
0.03
0.02
0.01

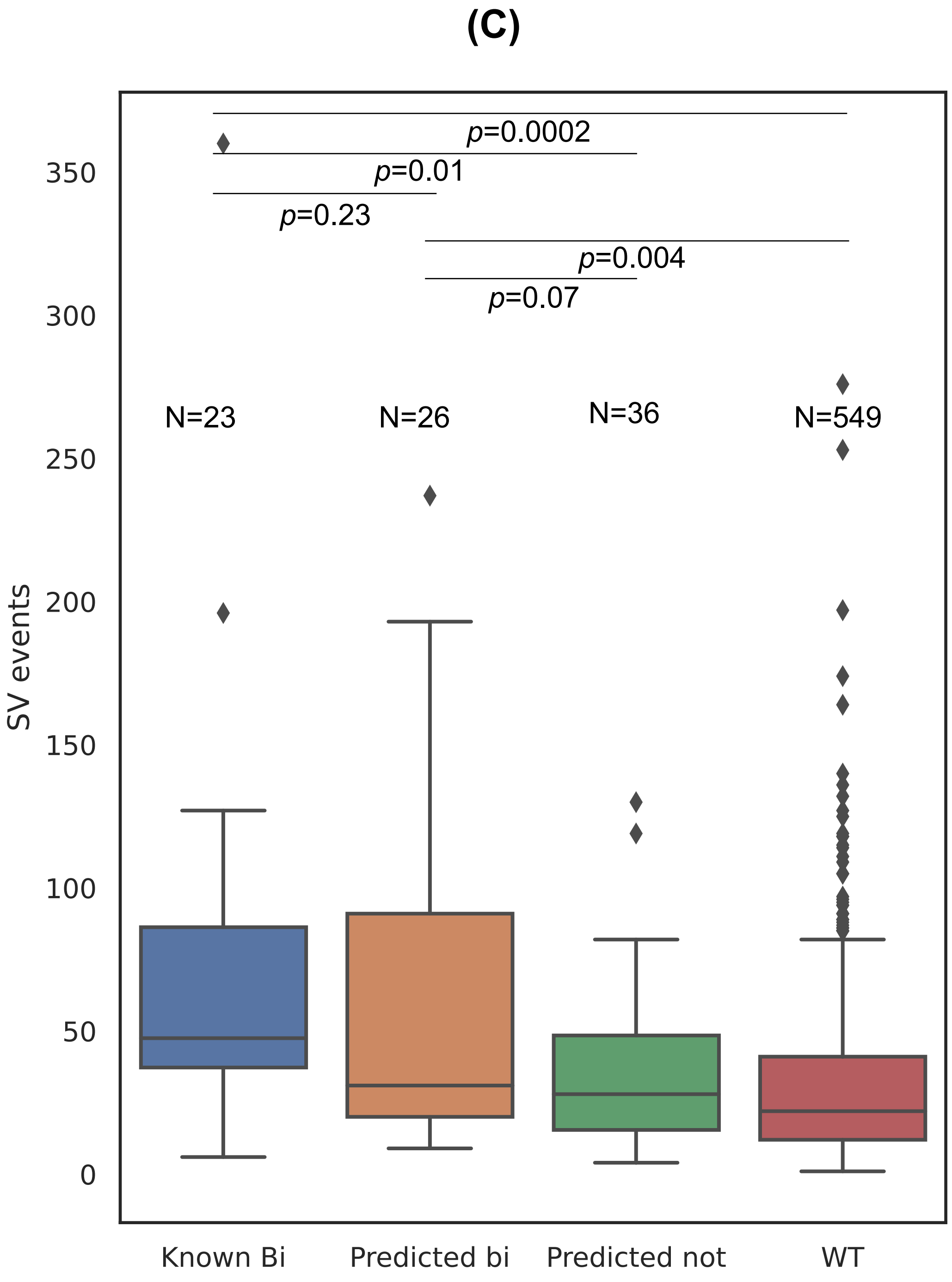

(C)
p=0.0002
p=0.01
p=0.23
p=0.004
p=0.07
N=23
N=26
N=36
N=549
SV events
0
50
100
150
200
250
300
350
Known Bi
Predicted bi
Predicted not
WT

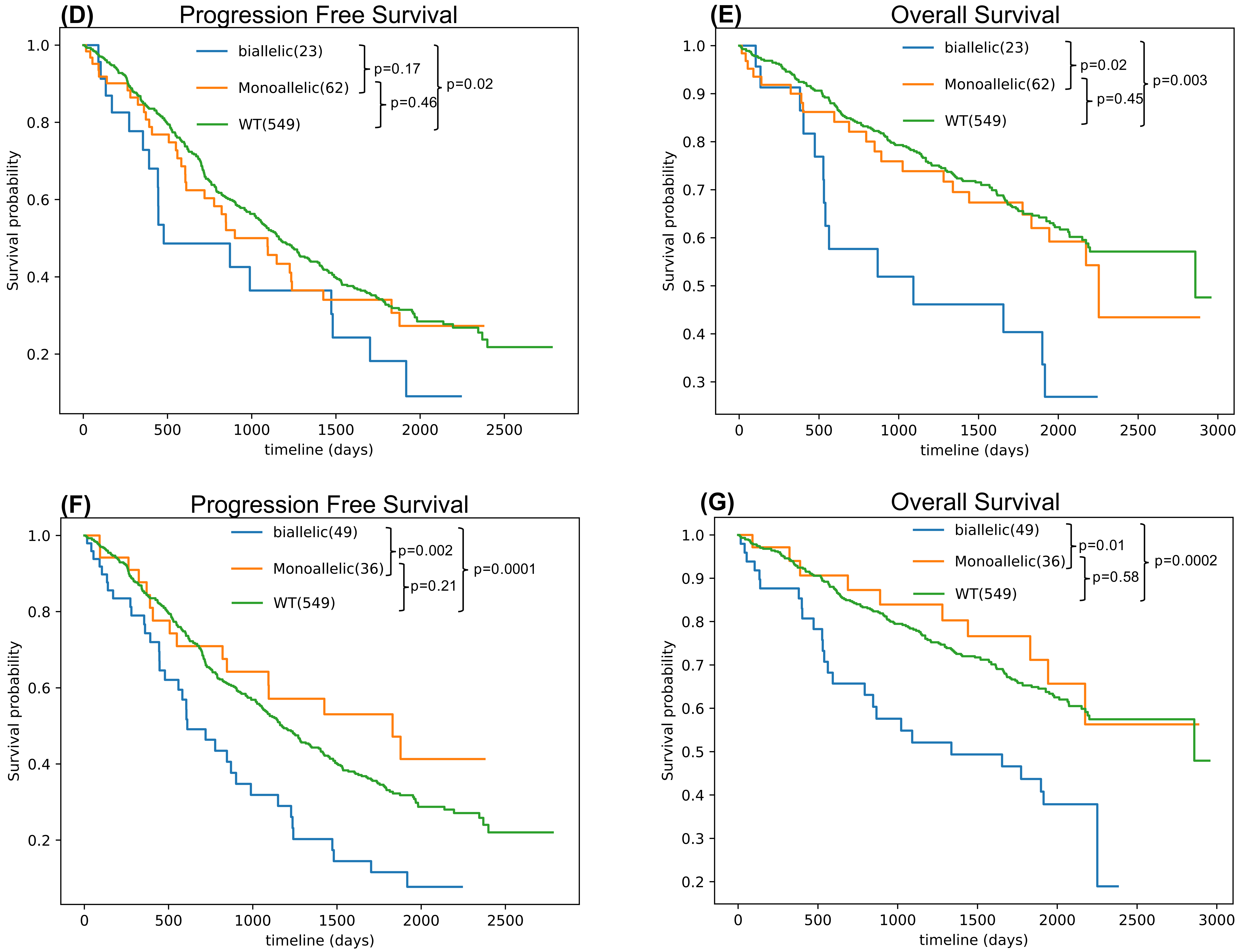

(D)
Progression Free Survival
biallelic(23)
Monoallelic(62)
WT(549)
p=0.17
p=0.46
p=0.02
Survival probability
timeline (days)
(E)
Overall Survival
biallelic(23)
Monoallelic(62)
WT(549)
p=0.02
p=0.45
p=0.003
Survival probability
timeline (days)
(F)
Progression Free Survival
biallelic(49)
Monoallelic(36)
WT(549)
p=0.002
p=0.21
p=0.0001
Survival probability
timeline (days)
(G)
Overall Survival
biallelic(49)
Monoallelic(36)
WT(549)
p=0.01
p=0.58
p=0.0002
Survival probability
timeline (days)

Supplementary Figure 3

Difference of durations between first diagnosis and relapse when biallelic *TP53* inactivation was initially discovered in RRMM patients.

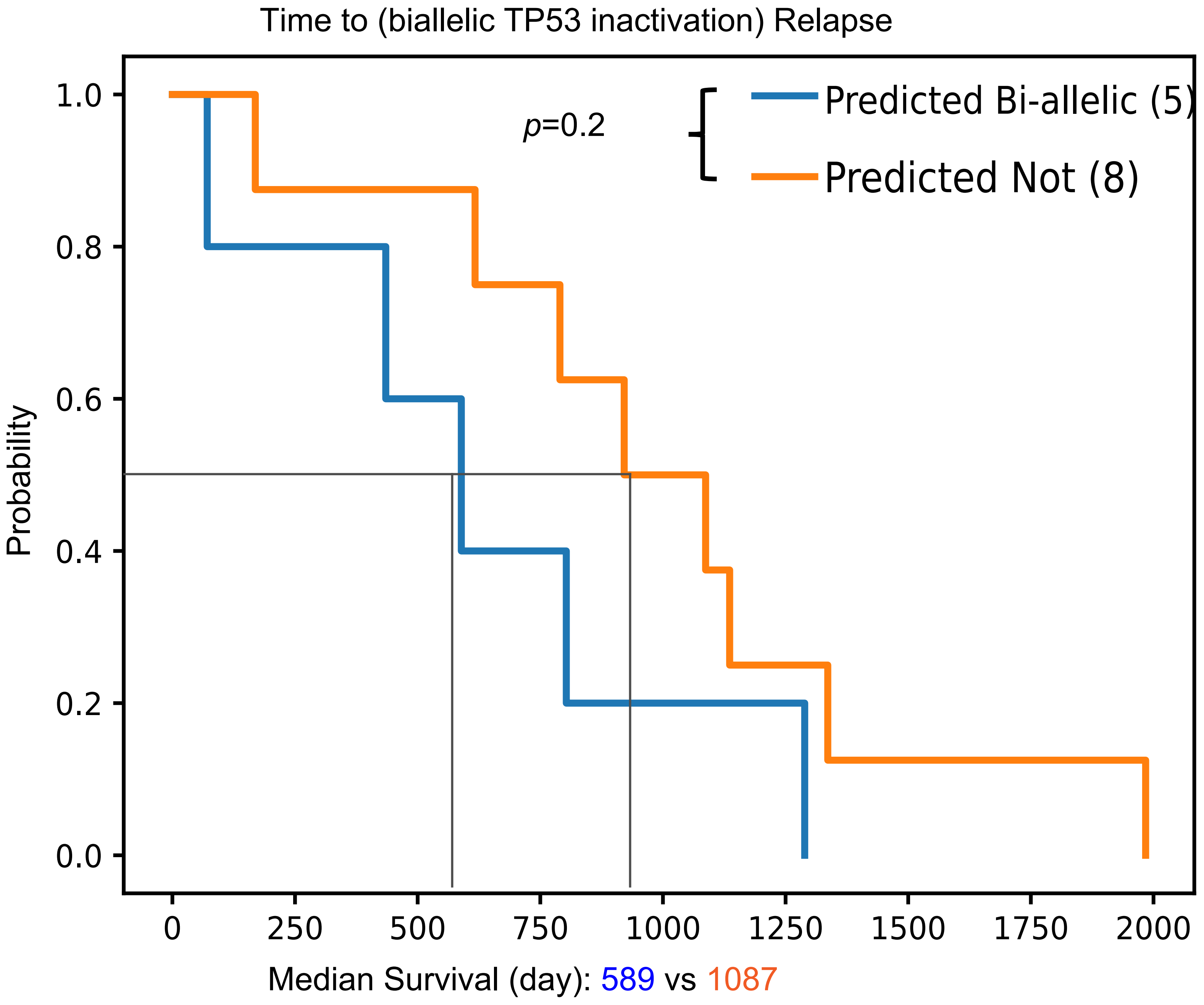

Supplementary Figure 4

Expression of *TP53* transcript variants across predicted biallelic samples and normal BMPCs. (**A**). Expression of full-length (TA) transcript variants. (**B**). Expression of$\Delta 40$ transcript variants. (**C**). Expression of $\Delta 133$ and $\Delta 160$ transcript variants. (**D**). Predicted translated peptide sequence from inferred novel transcript variant '*TP53*-v4' from **Fig. 3D**. Red peptides: start codon; Continuously highlighted peptide chains: Open read frames; (**E**). Ratio differences between expressed TAnormal/abnormal in a predicted biallelic sample and normal BMPCs.

(A)

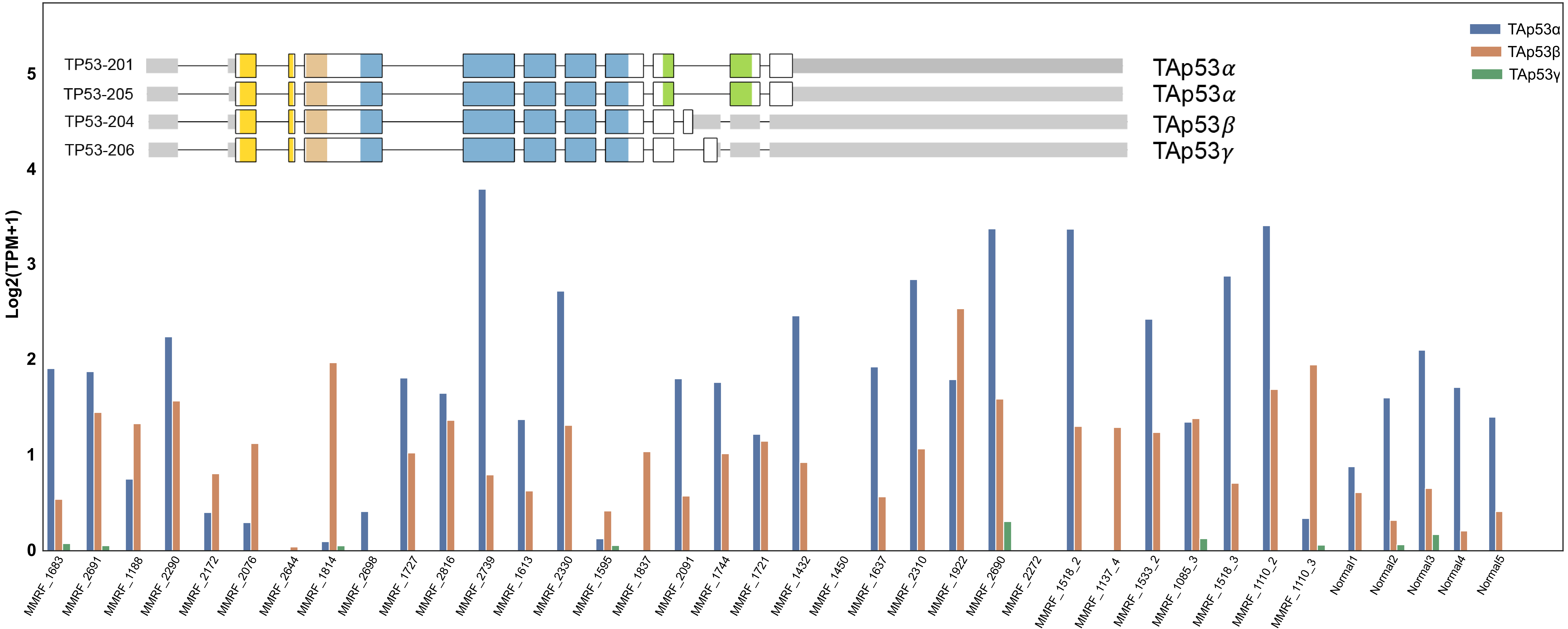

TP53-201
TP53-205
TP53-204
TP53-206
TAp53α
TAp53α
TAp53β
TAp53γ
TAp53α
TAp53β
TAp53γ
Log2(TPM+1)
0
1
2
3
4
5
MMRF_1683
MMRF_2691
MMRF_1188
MMRF_2290
MMRF_2172
MMRF_2076
MMRF_2644
MMRF_1814
MMRF_2698
MMRF_1727
MMRF_2816
MMRF_2739
MMRF_1613
MMRF_2330
MMRF_1595
MMRF_1837
MMRF_2091
MMRF_1744
MMRF_1721
MMRF_1432
MMRF_1450
MMRF_1637
MMRF_2310
MMRF_1922
MMRF_2690
MMRF_2272
MMRF_1518_2
MMRF_1137_4
MMRF_1533_2
MMRF_1085_3
MMRF_1518_3
MMRF_1110_2
MMRF_1110_3
Normal1
Normal2
Normal3
Normal4
Normal5

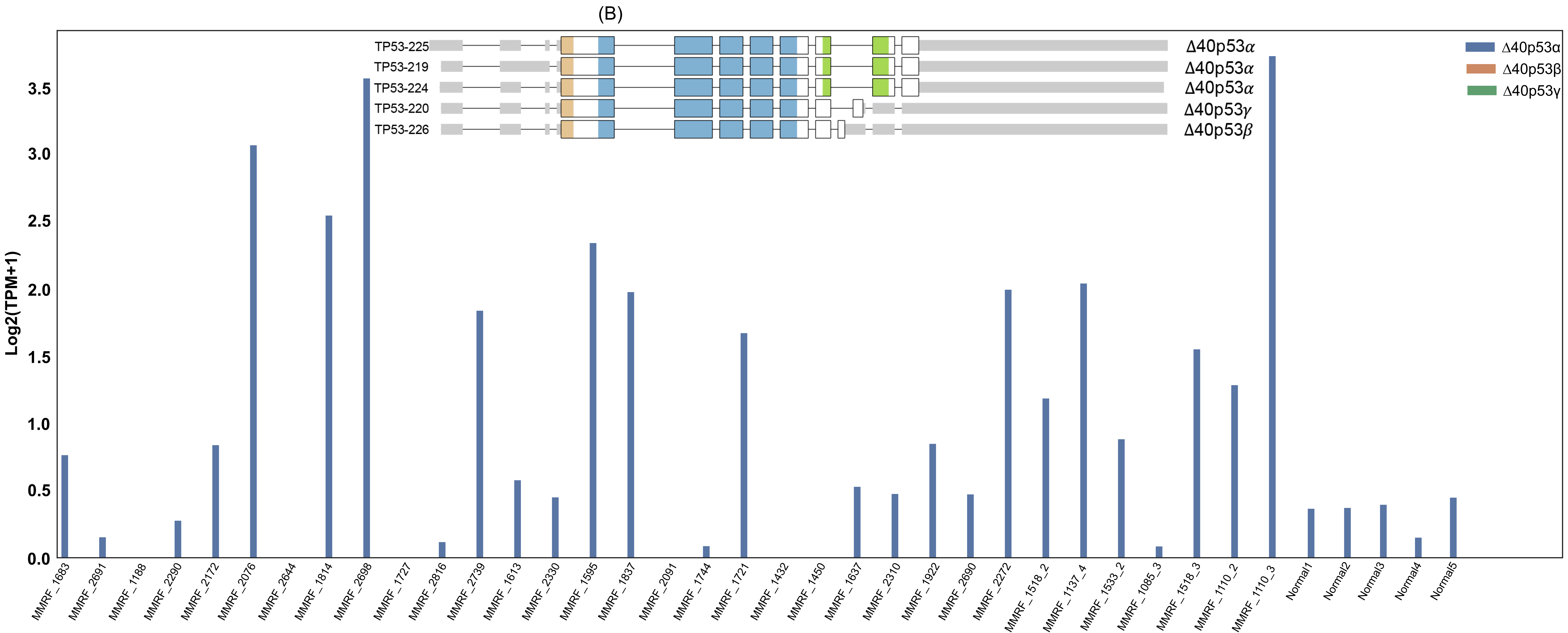

(B)
TP53-225
TP53-219
TP53-224
TP53-220
TP53-226
Δ40p53α
Δ40p53α
Δ40p53α
Δ40p53γ
Δ40p53β
Δ40p53α
Δ40p53β
Δ40p53γ
Log2(TPM+1)
0.0
0.5
1.0
1.5
2.0
2.5
3.0
3.5
MMRF_1683
MMRF_2691
MMRF_1188
MMRF_2290
MMRF_2172
MMRF_2076
MMRF_2644
MMRF_1814
MMRF_2698
MMRF_1727
MMRF_2816
MMRF_2739
MMRF_1613
MMRF_2330
MMRF_1585
MMRF_1837
MMRF_2091
MMRF_1744
MMRF_1721
MMRF_1432
MMRF_1450
MMRF_1637
MMRF_2310
MMRF_1922
MMRF_2690
MMRF_2272
MMRF_1518_2
MMRF_1137_4
MMRF_1533_2
MMRF_1085_3
MMRF_1518_3
MMRF_1110_2
MMRF_1110_3
Normal1
Normal2
Normal3
Normal4
Normal5

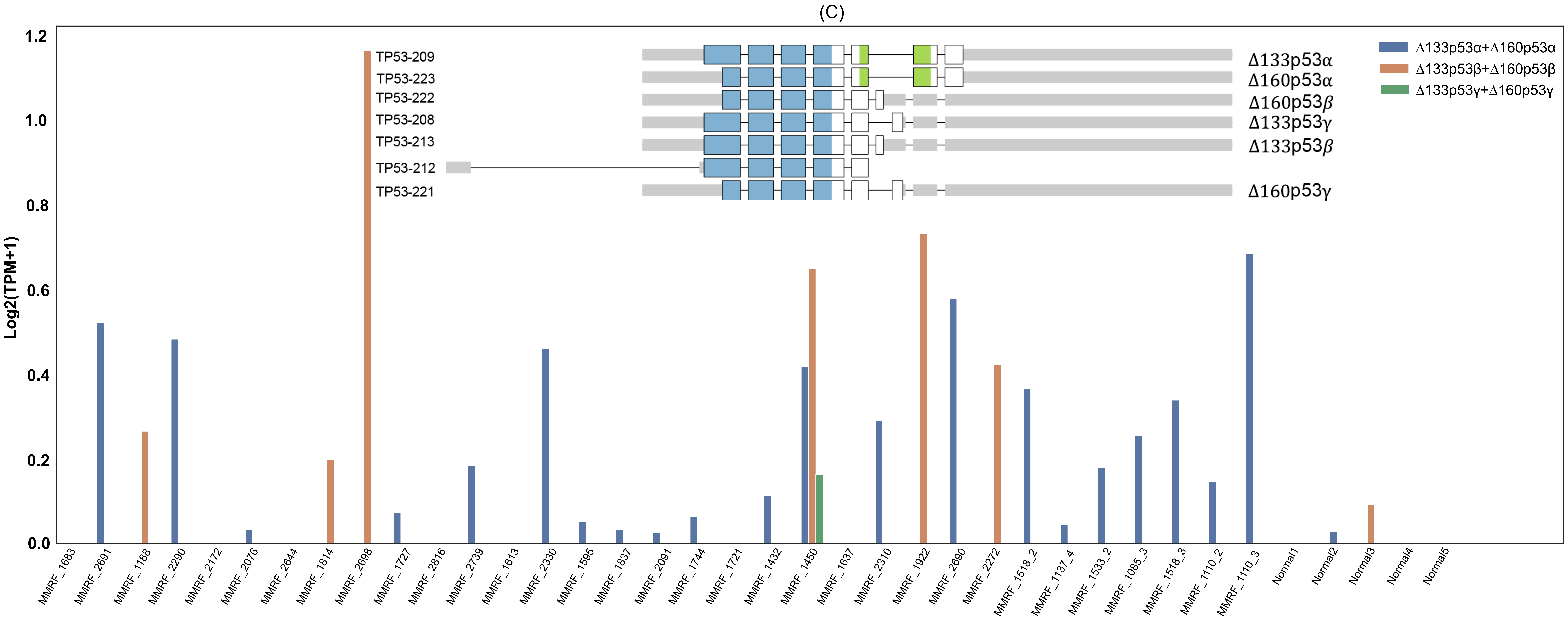
(C)
Log2(TPM+1)
TP53-209
TP53-223
TP53-222
TP53-208
TP53-213
TP53-212
TP53-221
Δ133p53α
Δ160p53α
Δ160p53β
Δ133p53γ
Δ133p53β
Δ160p53γ
Δ133p53α+Δ160p53α
Δ133p53β+Δ160p53β
Δ133p53γ+Δ160p53γ
MMRF_1683
MMRF_2691
MMRF_1188
MMRF_2290
MMRF_2172
MMRF_2076
MMRF_2644
MMRF_1814
MMRF_2698
MMRF_1727
MMRF_2816
MMRF_2739
MMRF_1613
MMRF_2330
MMRF_1595
MMRF_1837
MMRF_2091
MMRF_1744
MMRF_1721
MMRF_1432
MMRF_1450
MMRF_1637
MMRF_2310
MMRF_1922
MMRF_2690
MMRF_2272
MMRF_1518_2
MMRF_1137_4
MMRF_1533_2
MMRF_1085_3
MMRF_1518_3
MMRF_1110_2
MMRF_1110_3
Normal1
Normal2
Normal3
Normal4
Normal5

(D) *TP53*-v4

TAD domain TAD2 domain

VLSFSKV-SHRPGSR-
LLGSGDTLRSGWERAFHDGDTLPWIGSQTAFRVTAMEEPQSDPSVEPPLSQETFSDLWKLLPENNVLSPLPSQAMDDLMLSPDDIEQWFTEDPGPD
EAPRMPEAAPPVAPAPAAPTPAAPAPAPSWPLSSSVPSQKTYQGSYGFRLGFLHSGTAKSVTCTYSPALNKMFCQLAKTCPVQLWVDSTPPPGTRVRA
MAIYKQSQHMTEVVRRCPHHERCSDSDGLAPPQHLIRVEGNLRVEYLDDRNTFRHSVVVPYEPPEVWFATGVSGRRG- DBD domain
GWLSVALQVSSRGAFSCCLFDLPITP-DVQSKWV-LLHS-KN-SLQRLRASPAWLGAVAHACNPSTLGGQGRRITRLGDRDHPG-R-
NPVSTEKYKKKLAGRGAGHL-SQLLGRLRKENGVNLGGGACSELRSRHCTPAWATERDSISKKKKKGLPCLPQVSPRRTGLILGLCYLLGWL-
LYHHPLQLHV-QFLHGRHEPEAHPHHHHTGRLQVRSHLPPCTLACCAPASACL-
PLGPPLTDFFHTTTHPPLITSPAGNLLTAPTQFSFLWLWDLLTCGFSSTYLELELRLQKGQGWLGVDGAWFFKWDR-DLISLLPLASLFLS-
VVVIYWDGTALRCVFVPVLGETGAQRKRISARKGSLTTSCPQGALSEHCPTTPAPLPSQRRNHWMENISPFRY-VLGPLIKWKVSSLTLKMPFSS-
LFYLQLGHLPSGGSDASKTMAPGCS-LTSEHQLIP-YIF-RTRPAFKKKIVKESMKMVL-LCLIQMLLDLRWCYFLINSS- γ Suffix
VENIIRGRERFEMFRELNEALELKDAQAGKEPGGSRAHSSHLKSKKGQSTSRHKKLMFKTEGPDSD-HSPLLVPH-QPPTPISPSPAILGFGSLNPCLQ-
VCVRSTQDFHLLCPGAPLNKLACTGVLLWGGGWGVGHTSLDFKVFTVRDVWEM-EMFLQLRVSLQSATF-VGAHFTVLTREAVPHC-
IFSNFKAHICEMLAFAPTSQSAL-GLMK-CTSGLETTFYYMGSRT-PP-GCLFPLPVGRWVGSFYSWAAG-VEGVVKSLLAQPNPV-QPLGEP-
YLKGNLTPSHTLEDFISCI-- Tetramerization domain
SGSTKTCFMLRVNFFFLFFFFFSFSLRLGLALLPRLEWSGVILAYCSLCLPGSSSPASASGVAGTTGSCHHGQPTFACFVEMGSHSVAQAGLKLLGSGDPP
VSASQSAGITIVSHHVQLEGSTSFTFCKHICIFTPPFPSFSLFISHFYIDLLFYNKTLLP

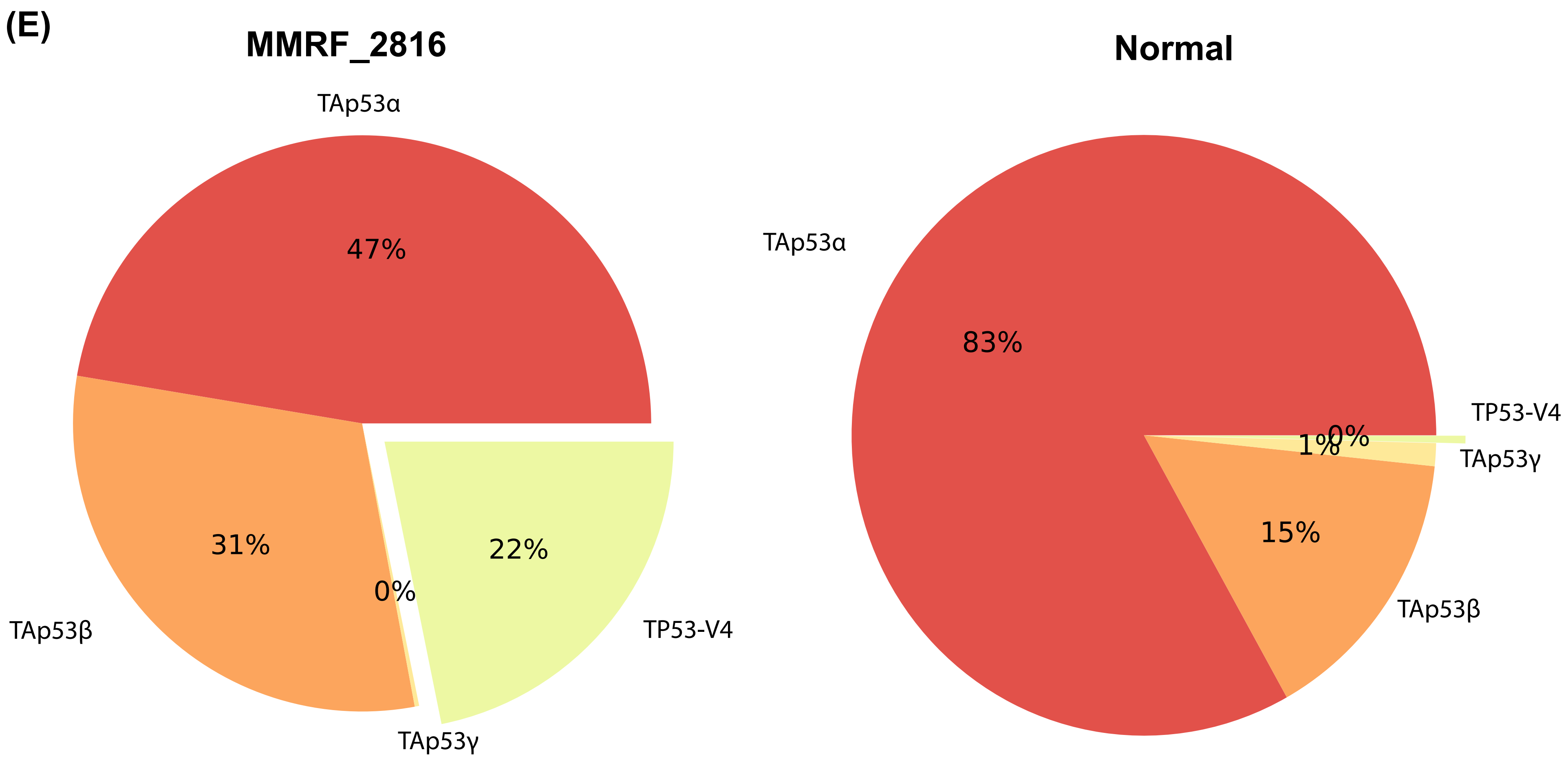
(E)
MMRF_2816
TAp53α
47%
31%
0%
22%
TAp53β
TP53-V4
TAp53γ
Normal
TAp53α
83%
TP53-V4
1%
0%
TAp53γ
15%
TAp53β

Supplementary Figure 5

Sashimi plots of the splice site mutations found in MMRF_1814 (**A**), MMRF_1915 (**B**), MMRF_1991 (**C**).

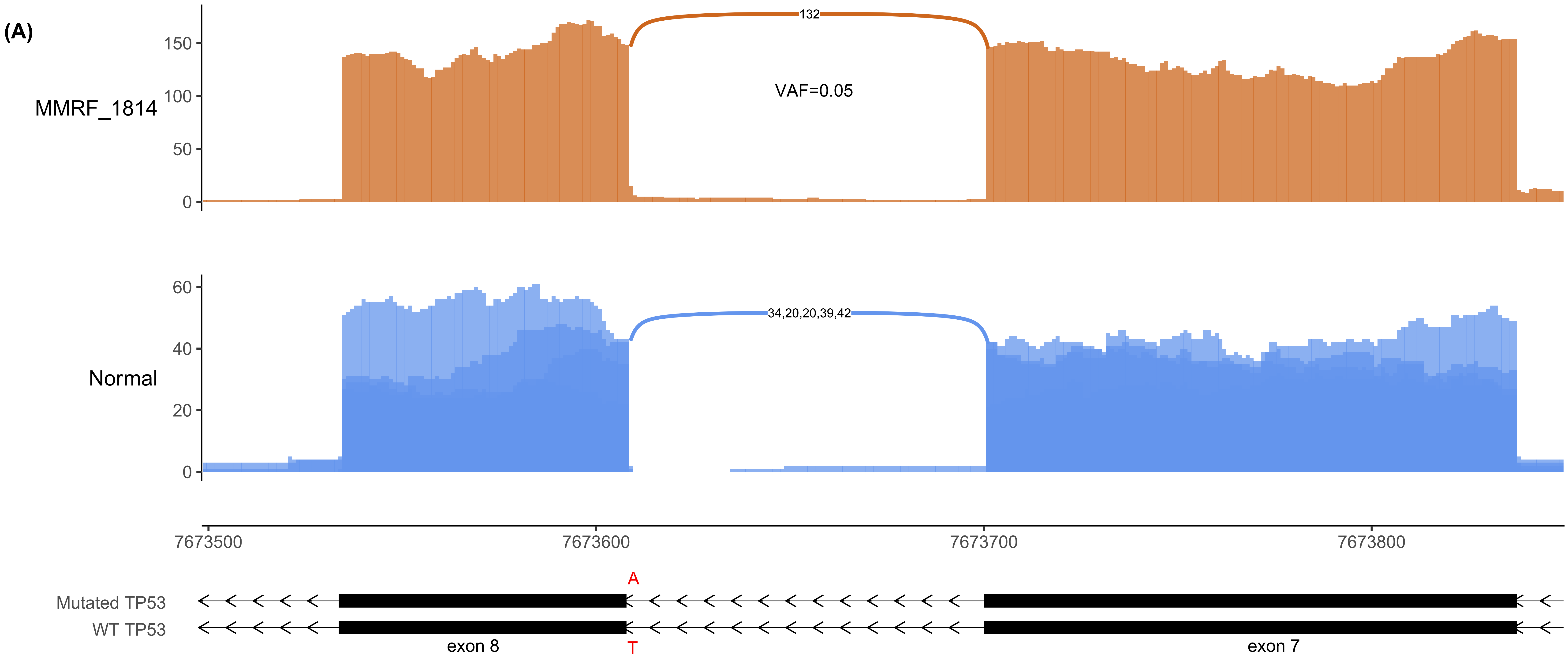

(A)
MMRF_1814
132
VAF=0.05
150
100
50
0
Normal
34,20,20,39,42
60
40
20
0
7673500
7673600
7673700
7673800
A
Mutated TP53
WT TP53
T
exon 8
exon 7

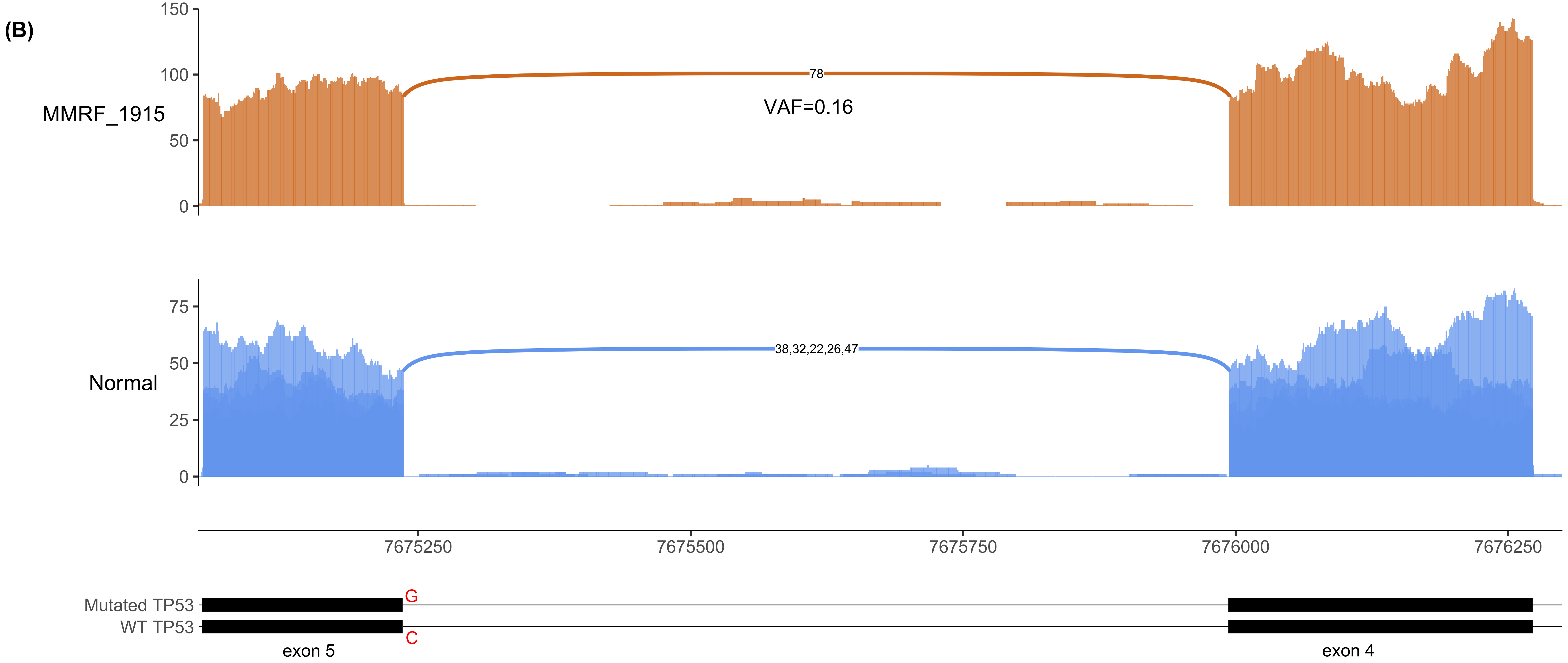
(B)
MMRF_1915
150
100
50
0
78
VAF=0.16
Normal
75
50
25
0
38,32,22,26,47
7675250
7675500
7675750
7676000
7676250
Mutated TP53
WT TP53
G
C
exon 5
exon 4

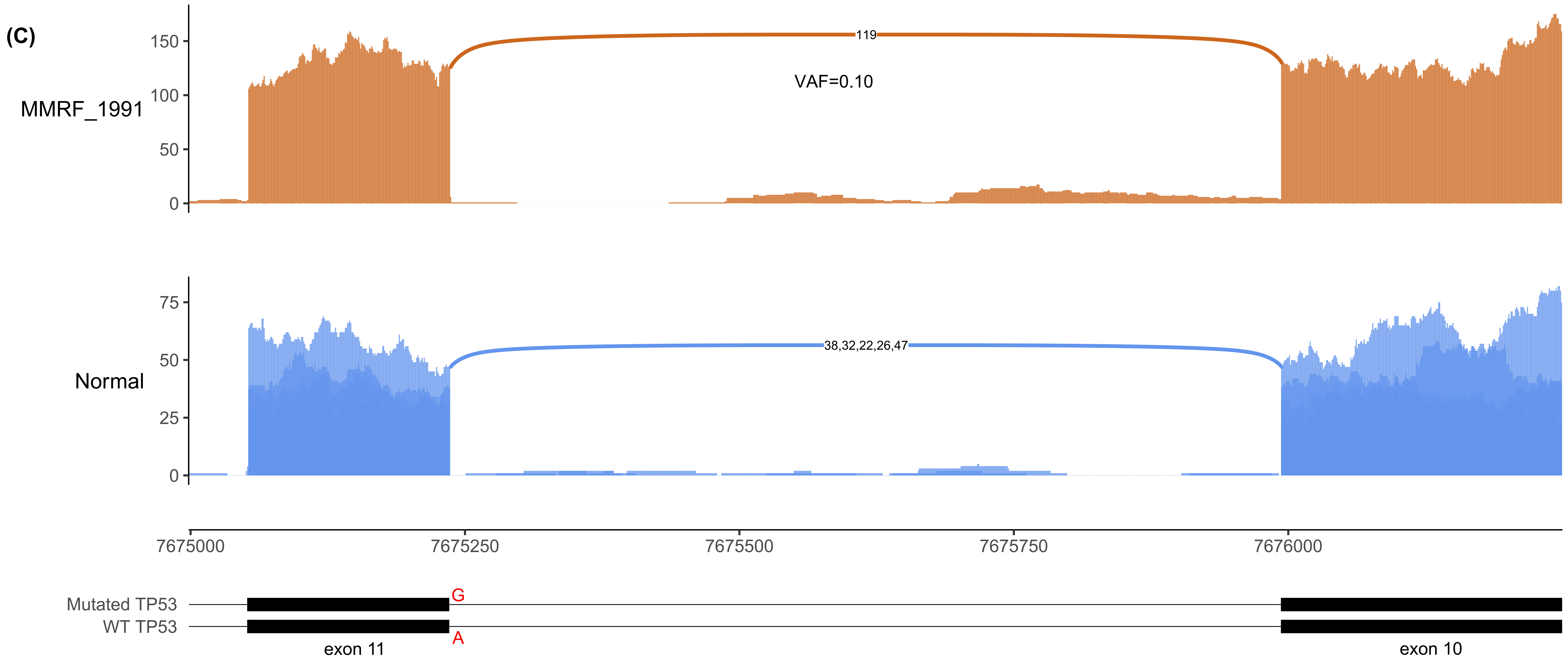
(C)
MMRF_1991
150
100
50
0
119
VAF=0.10
Normal
75
50
25
0
38,32,22,26,47
7675000
7675250
7675500
7675750
7676000
Mutated TP53
WT TP53
G
A
exon 11
exon 10

Reference

1. Liao,Y.,et al., *WebGestalt 2019: gene set analysis toolkit with revamped UIs and APIs***.** 2019. , 47(W1):W199-W205
2. Zhan, F., et al., *The molecular classification of multiple myeloma.* 2006. **108**(6): p. 2020-2028
